# Association of genetic variants in *ULK4* with the age of first onset of type B aortic dissection

**DOI:** 10.3389/fgene.2022.956866

**Published:** 2022-09-02

**Authors:** Lihong Huang, Jiaqi Tang, Lijuan Lin, Ruihan Wang, Feng Chen, Yongyue Wei, Yi Si, Weiguo Fu

**Affiliations:** ^1^ Department of Vascular Surgery, Zhongshan Hospital, Fudan University, Shanghai, China; ^2^ Department of Biostatistics, Zhongshan Hospital, Fudan University, Shanghai, China; ^3^ Clinical Research Unit, Institute of Clinical Science, Zhongshan Hospital of Fudan University, Shanghai, China; ^4^ Department of Biostatistics, School of Public Health, Nanjing Medical University, Nanjing, China

**Keywords:** type B aortic dissection, *ULK4*, age first onset, genetic variants, gene expression

## Abstract

**Background:** The association between autophagy, structural alterations of the aortic wall, and endothelial dysfunction in humans has yet to be fully elucidated. The family of *ULK* (UNC51-like) enzymes plays critical roles in autophagy and development. This study aimed to evaluate the association between *ULK* gene family members and patient age of first type B aortic dissection (TBAD) onset.

**Methods:** The genotype data in a TBAD cohort from China and the related summary-level datasets were analyzed. We applied the sequence kernel association test (SKAT) to test the association between single-nucleotide polymorphisms (SNPs) and age of first onset of TBAD controlling for gender, hypertension, and renal function. Next, we performed a 2-sample Mendelian randomization (MR) to explore the potential causal relationship between *ULK4* and early onset of TBAD at the level of gene expression coupled with DNA methylation with genetic variants as instrumental variables.

**Results:** A total of 159 TBAD patients with 1,180,097 SNPs were included. Concerning the association between the *ULK* gene family and the age of first onset of the TBAD, only *ULK4* was found to be significant according to SKAT analysis (q-*FDR* = 0.0088). From 2-sample MR, the high level of *ULK4* gene expression was related to a later age of first onset of TBAD (*β* = 4.58, *p* = 0.0214).

**Conclusion:** This is the first study of the *ULK* gene family in TBAD, regarding the association with the first onset age. We demonstrated that the *ULK4* gene is associated with the time of onset of TBAD based on both the SKAT and 2-sample MR analyses.

## Introduction

Type B aortic dissection (TBAD) is a rare while life-threatening condition, in which a tear occurs in the descending part of the aorta and may extend into the abdomen (Prêtre and Von Segesser, 1997; Hagan et al., 2000; Nienaber and Clough, 2015). Prevention of premature death from TBAD depends on the early identification of high-risk individuals, careful monitoring of the dissected aorta for aneurysmal dilations, medications to slow the rate of growth of aneurysms, and timely surgical repair of aneurysms (Mokashi and Svensson, 2019).

Aortic expansion is one of the risk factors associated with the need for intervention or adverse outcomes in patients with TBAD. It was reported that younger age at presentation was a clinical predictor of aortic expansion. Patient age <60 years was significantly associated with increasing aortic diameter, which was thought to be due to a less rigid aortic wall, making the aorta more prone to dilation in younger patients (Kamman et al., 2017). However, the essential reasons for this finding deserve further research, and the association of genetic variants with the age of first onset of type B aortic dissections is a valuable research direction.

Autophagy is a process in which intracellular components and dysfunctional organelles are delivered to the lysosome for degradation and recycling. Therefore, autophagy has various connections to many human diseases, as its functions are essential for cell survival, bioenergetic homeostasis, organism development, and cell death regulation. The association between autophagy and structural alterations of the aortic wall and endothelial dysfunction in humans has yet to be fully elucidated (Yang and Klionsky, 2020). Previous studies have shown that more than 20% of individuals with thoracic aortic aneurysms and dissections have a family history of disease that may be caused by a genetic syndrome, resulting from a single-gene mutation such as Marfan syndrome (MFS [MIM:154700]) arising from a fibrillin-1 (FBN1 [MIM:134797]) mutation (Biddinger et al., 1997). However, autophagy-related biomarkers studies for aortic dissection diseases are still rare. According to existing research, AMPK increases the process of autophagy after its activation. Although the mechanisms of AD and autophagy have not been fully elucidated, autophagy has been observed to be activated in impaired vascular smooth muscle cells (VSMCs). Excessive or impaired autophagy may lead to VSMC death or dysfunction, which is thought to promote aneurysm and AD (Clément et al., 2019).

It is known that the *ULK* (UNC51-like) enzymes are a family of mammalian kinases and play critical roles in autophagy and development. The mammalian *ULK* family of kinases comprises 5 genes: *ULK1* to *ULK4* and *STK36* (Chan and Tooze, 2009). These enzymes share a conserved N-terminal kinase domain, which is homologous to *C. elegans* UNC51 and yeast Atg1, the original kinase identified in the autophagy pathway. *ULK* kinases can be found in all observed eukaryotes. *ULK1* kinases are involved in autophagy (Young et al., 2009), *ULK3* is also implicated in hedgehog signaling and in autophagy-mediated senescence (Maloverjan et al., 2010), and several genome-wide association studies (GWASs) show linkage to blood pressure (Levy et al., 2009). *ULK4* is a pseudo kinase in all species and may be linked to neurogenesis, brain function (Liu et al., 2017), and blood pressure (Levy et al., 2009). It has also been reported that *ULK4* is potentially associated with acute aortic dissections (Guo et al., 2016). As can be seen, there is demonstrable link between the *ULK* family, autophagy, and acute aortic dissection, and thus an assessment of the effect of the *ULK* family on aortic dissection is warranted.

We performed an association analysis between *ULK* gene family members and the age of first onset of Chinese TBAD patients. The association study was performed in Chinese TBAD patients. The causal effect of *ULK* genes was subsequently verified by 2-sample Mendelian randomization (MR) at the gene expression and DNA methylation levels.

## Materials and methods

### Study population and data source

We obtained genotype data from a TBAD cohort enrolled through the Vascular Surgery Department of Zhongshan Hospital Fudan University, which included 162 Chinese patients with TBAD from January 2018 to June 2019. Each participant signed a consent form. The study was approved by the relevant ethics committees (Ethical approval No. B2019-110R) and was administered by trained personnel.

### Genotyping and quality control of whole genome sequencing data

In association analysis, we obtained genotype data using whole genome sequencing, using the Illumina NovaSeq platform (Illumina, San Diego, CA, United States) in a paired-end 150 bp mode on 162 TBAD patients. Briefly, the samples were excluded if they met any of the following quality control (QC) criteria (Supplementary Figure S1): 1) overall genotype completion rate <95%, 2) unexpected duplicates or probable relatives, 3) heterozygosity rates more than six times the SD from the mean, or 4) gender discrepancies. SNPs were excluded if they met any of the following QC criteria: 1) SNPs had a low call rate of <95% in all samples, 2) the genotype distributions of SNPs deviated from those expected by the Hardy–Weinberg equilibrium (*p* < 0.000001), or 3) single-nucleotide variants (SNVs) with minor allele frequencies were less than 1%.

### Summary-level data of expression quantitative trait loci and methylation quantitative trait loci

Methylation quantitative trait loci (mQTL) data were obtained from the Brisbane Systems Genetics Study (*n* = 614) and Lothian Birth Cohorts of 1921 and 1936 (*n* = 1366) (Wu et al., 2018). Details of the QC procedures were described in a previous study. Briefly, all the individuals were of European descent. Only the DNA methylation probes with at least a cis-mQTL at *p* < 0.0001 and only SNPs within 500 kb distance from each probe were included in the analysis. As for the summary statistics of expression quantitative trait loci (eQTL), we used the cis-eQTL in the prefrontal cortex from the PsychENCODE project (*n* = 1387) (Gandal et al., 2018; Wang et al., 2018). The eQTL analyses of PsychENCODE were performed by including 100 hidden covariate factors as covariates. Only the data of SNPs in a 500 kb window around the *ULK4* gene were included in the subsequent analysis.

### Statistical analysis

Continuous variables are summarized as mean ± SD and categorical variables are described as numbers and percentages. The sequence kernel association test (SKAT), which is a supervised, flexible, and computationally efficient regression method was used to test for the association between a set of genetic variants and a continuous or dichotomous trait with adjustments made for relevant covariates (Ionita-Laza et al., 2013). A total of 421 variants from the *ULK* family passed QC and were included in SKAT analysis with controlling for gender, hypertension, and smoking status and renal function (Wu et al., 2011). Furthermore, separate analyses were conducted for all variants (*n* = 421) and rare variants with minor allele frequency (MAF) < 0.05 (*n* = 290). We used COXPRESdb (http://coxpresdb.jp) to drawing the co-expressed gene network with pathway and protein–protein interaction information. COXPRESdb was first released for human and mouse models in 2007 (Takeshi et al., 2007). One characteristic feature of COXPRESdb is its ability to compare multiple co-expression data derived from different transcriptomics technologies and different species, which strongly reduces false-positive relationships in individual gene co-expression data (Takeshi et al., 2012; Takeshi et al., 2019). To clarify the molecular mechanisms, we used COXPRESdb to perform co-expression analysis on those genes that exerted significant effect on aortic dissection.

**FIGURE 1:**
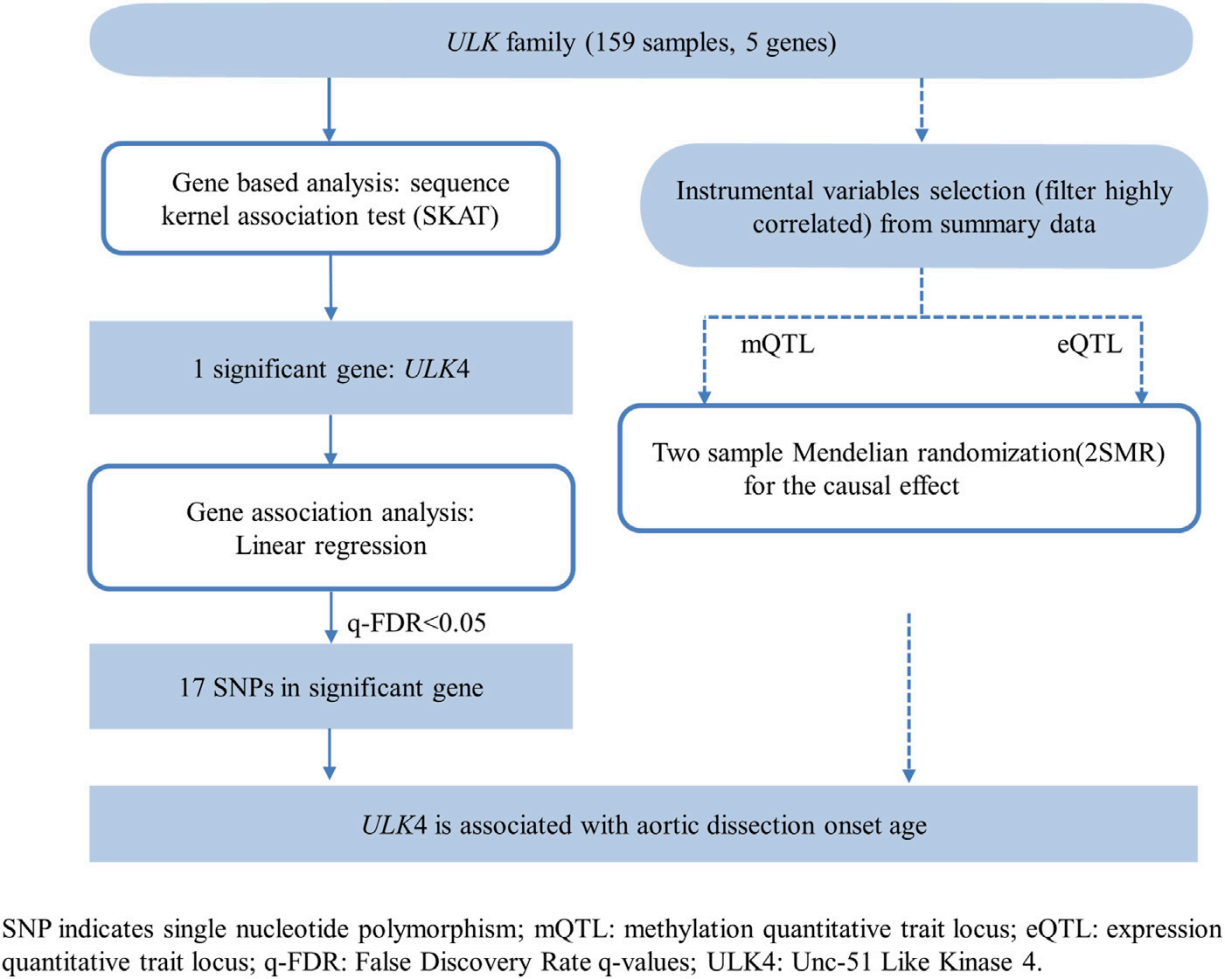
Flow chart of the study design and statistical analysis.

The analysis workflow is shown in Figure 1. In general, we adopted two analysis steps. First, we applied the SKAT to test the association between a set of SNP and patients’ age of first onset of TBAD, controlling for gender, hypertension, smoking status, and renal function. Then, linear regression models were applied to further detect significant SNPs in significant genes. Multiple comparisons were adjusted with the false discovery rate method (FDR) to control the overall false-positive rate at a 5% level. Biomarkers measured by a q-FDR value ≤0.05 were included in further study (Benjamini and Hochberg, 1995).

To further explore the role of *ULK4* in different clinical features, we constructed a genetic score with the significant SNPs and conducted subgroup analysis to compare the differences between different categories of features. The genetic score (GS) was calculated based on a weighted linear combination of individual values of the significant SNPs, with weights derived from the stepwise linear regression model. As a result, three SNPs were finally included in the genetic score with the formula defined as follows: GS = (−25.326 × rs191792955) + (12.408 × rs142574024) + (−4.425 × rs74282513).

In the second step, we performed a 2-sample MR to explore the potential causal relationship between *ULK4* and the early onset of TBAD (*Y*) at the level of gene expression and DNA methylation (*X*) using genetic variants (*G*) as instrumental variables (Evans and Davey Smith, 2015). As shown in Figure 4, SNPs with a q-FDR < 0.05 were regarded as candidate instrumental variables and linkage disequilibrium (LD) clumping with a window of 1 MB, and an *r* ^*2*^ < 0.2 was applied to remove SNPs with high LD (Hemani et al., 2018). In addition, we performed sensitivity analyses using several approaches to investigate potential pleiotropic bias and verify the robustness of the results, including MR-Egger regression, weighted median MR, weighted mode MR, simple mode MR, funnel plots, and leave-1-variant-out analysis.

Statistical analyses were performed using R version 3.4.4 (The R Foundation of Statistical Computing, Vienna, Austria). A 2-sided *p*-value of less than 0.05 was considered to indicate a statistically significant difference.

## Results

A total of 162 Chinese TBAD patients from Zhongshan Hospital were genotyped with two patients being excluded because of gender mismatches, and one patient being excluded because of familial relationships. Finally, 159 TBAD patients with 1,180,097 SNPs after QC were included in further analyses (Supplementary Figure S1). Demographics and clinical characteristics are shown in Supplemetary Table S1. The mean age of the enrolled TBAD inpatients was 56.11 years (SD 14.33), and 78.62% of the patients were male. The mean age of the first onset of TBAD was 54.89 years with a range of 18–85 years. Most of the enrolled TBAD inpatients (88.68%) also had hypertension, and 10.06% had diabetes.

**FIGURE 2:**
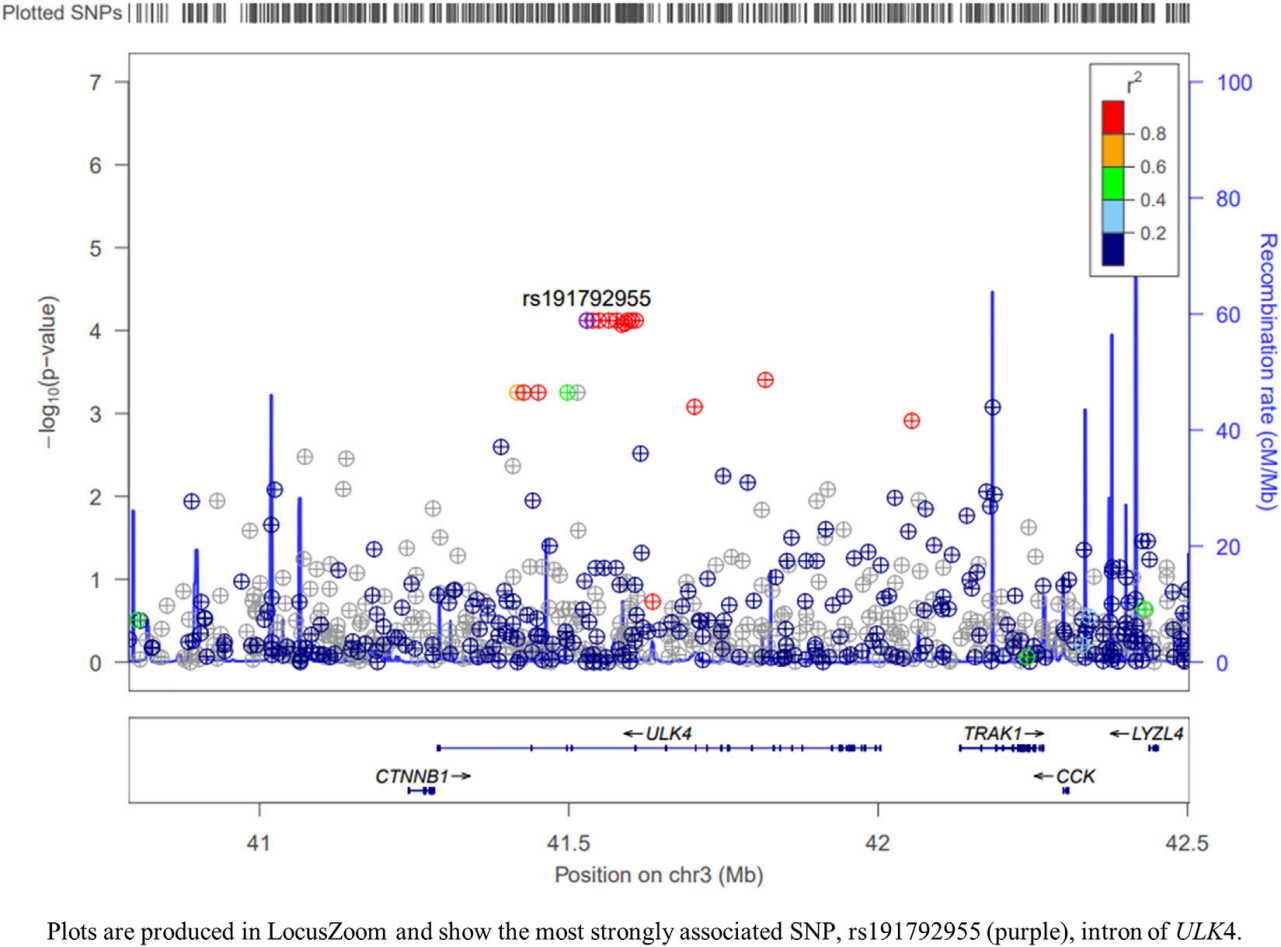
Regional association plots for ULK4.

Concerning on the association between the *ULK* gene family and age of first onset of TBAD, only *ULK4* was significant according to SKAT analysis (q-*FDR* = 0.0088) based on our TBAD samples. Among 361 variants in the *ULK4* gene, there were 243 variants with MAF <0.05. In addition, SKAT analysis for variants with MAF <0.05 of *ULK4* was also significant (*p* = 0.0015) (Table 1). Further linear regression analysis of SNPs in *ULK4* revealed 17 SNPs, that were associated with age of first onset of TBAD, and the most strongly associated SNP in *ULK4* was rs191792955 (Figure 2; Supplementary Figure S2). The details of the association results of the SNPs sites of *ULK4* are shown in Supplementary Table S2.

**TABLE 1:** Results of the ULK family regional gene-based SKAT analysis for aortic dissection.

Candidate gene	All variants			MAF < 0.05		
	Number	*p*-value	q-FDR	Number	*p*-value	q-FDR
ULK1	18	0.7312	0.7312	13	0.6788	0.6788
ULK2	40	0.2331	0.5828	32	0.2127	0.5317
ULK3	2	0.6032	0.7312	2	0.6032	0.6788
ULK4	361	0.0018	0.0088	243	0.0015	0.0074
STK36	10	0.5855	0.7312	6	0.5156	0.6788

Models adjusted for sex, hypertension controlling, and renal function. Data source: TBAD cohort.

Among the significant 17 SNPs, three SNPs were maintained for genetic score generation after stepwise regression with *p* < 0.05. As the genetic score increased, the age of first onset decreased for TBAD patients with well-controlled hypertension (coefficient 1.21, 95% CI: 0.677–1.751), and similar results were found for complex, male, and normal renal function TBAD patients. Heterogeneity details are shown in a forest plot (Supplementary Figure S3).

**FIGURE 3:**
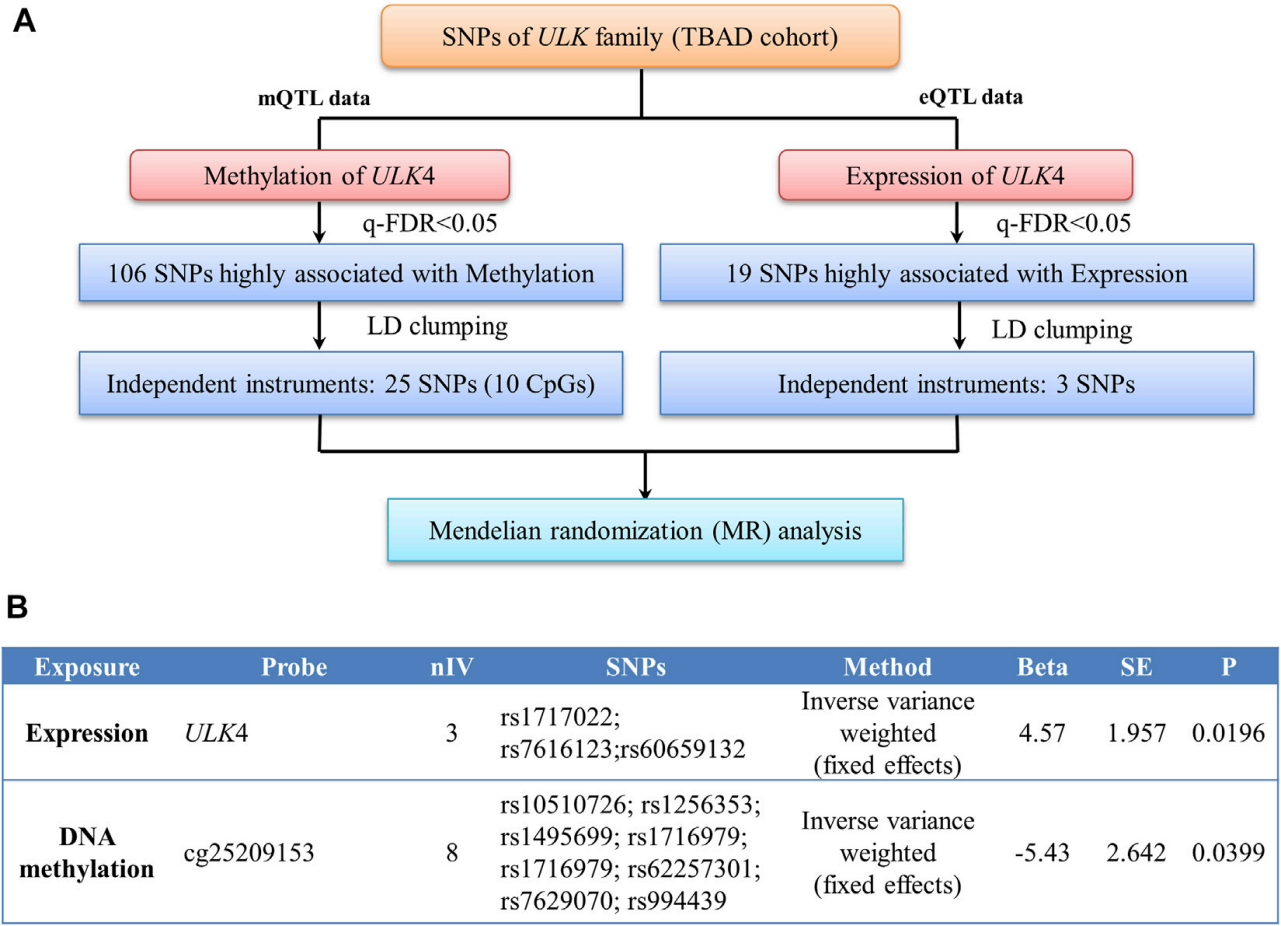
Two sample Mendelian randomization analyses. **(A)** Diagram of instrumental variable (IV) selection. **(B)** Results of Mendelian randomization (MR) between gene expression, DNA methylation, and onset age of TBAD.

**FIGURE 4:**
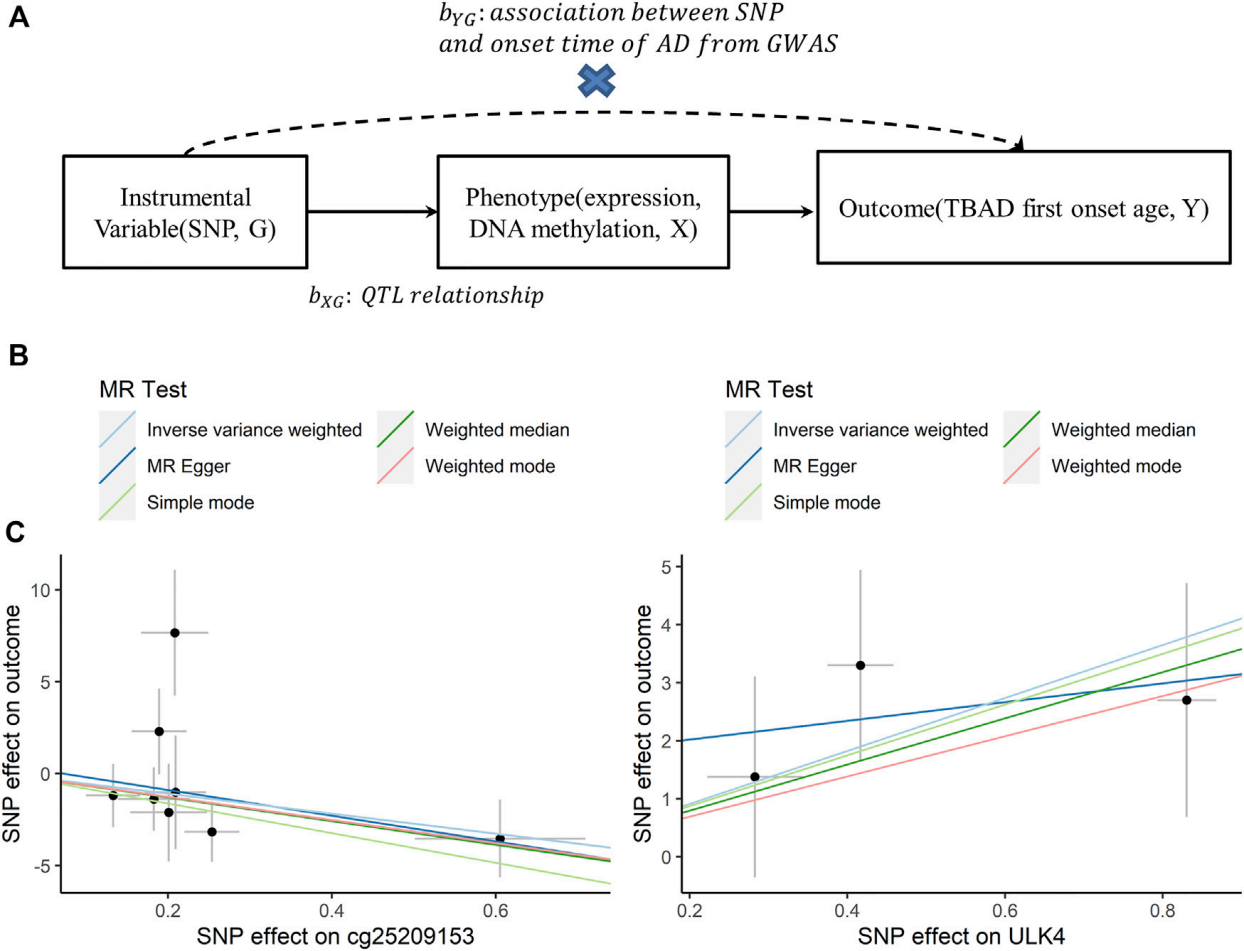
Association plots between ULK4 or cg25209153 and first onset age. **(A)** Diagram of Mendelian randomization (MR) analysis. **(B)** Scatter plots for association between cg25209153 and first onset age by different MR analytical methods. **(C)** Scatter plots for association between ULK4 and first onset age by different MR analytical methods.

For 2-sample MR, the individual instrument-gene expression and instrument-age of first onset for TBAD are shown in Supplementary Table S3. Among the 19 SNPs associated with gene expression (q-FDR < 0.05), three were left after LD clumping. A similar process was followed for the instrumental variable determination of *ULK4* methylation, and there were 10 CpG probes with 25 SNPs found (Supplementary Table S4). The details of the instrumental variable selections are shown in Figure 3A. The intercept of MR-Egger for *ULK4* gene expression and DNA methylation indicated that there was no potential horizontal pleiotropy, and the inverse variance weighted (IVW) analysis with fixed effects was conducted for two sample MR analyses. The high *ULK4* gene expression was related to a later age of onset for TBAD (*β* = 4.58, *p* = 0.0214) (Figures 3B, 4A). Among MR analyses of the 10 CpG probes, eight SNPs in cg25209153 were significant (*p* = 0.0155) (Figures 3B, 4A), and the higher cg25209153 was related to the earlier age of first onset (*β* = −4.02). In addition, several MR methods were used to evaluate the robustness of results, and the direct of the causal effects of both *ULK4* and cg25209153 were consistent with IVW, although the *p* values were not significant (Figures 4B, C). The details of sensitively analyses are shown in Supplementary Table S5.

According to the gene co-expression network of *ULK4* (Supplementary Figure S4), it was found that OSCP1 acts on smooth muscle cells in the tunica media layers of artery walls, participates in the regulation of the extracellular matrix (ECM) related to intimal proliferation after endothelial injury, and is related to restenosis after vascular injury (Martí‐Pàmies et al., 2017). WDPCD is believed to be related to the development of arteries. In the process of coronary vasculature development, WDPCD participates in the regulation of epithelial–mesenchymal transition (EMT) to enable migration that gives rise to smooth muscle cells (Liu et al., 2018).

## Discussion

Acute aortic dissection may be fatal without early diagnosis and appropriate management, and thus biomarker tests play an important role in preventing and diagnosing aortic dissection disease (Ranasinghe and Bonser, 2010). Several epigenetic studies of TBAD have identified potential biomarkers relevant to the etiology of TBAD (Wang et al., 2012; Erhart et al., 2020). Multiple GWAS studies have identified a significant association of the *ULK4* SNPs with hypertension. Genetic variants in *ULK4* have also been reported to be associated with the pathogenesis of sporadic thoracic aortic dissection (STAD) (Guo et al., 2016). To the best of our knowledge, this is the first study of the *ULK* gene family in TBAD to focus on the association with age of first onset.

The *ULK* (UNC51-like) enzymes play critical roles in autophagy and development. While *ULK1*, *ULK2*, and *ULK3* have been characterized, and very little is known about *ULK4*. Recently, deletions in *ULK4* have genetically linked to increased susceptibility to developing schizophrenia, which is a devastating neuropsychiatric disease with high heritability (Khamrui et al., 2019). Similarly, TBAD has also been identified as a suspected heritable characteristic. In their single-institute study, Shalhub et al. (2021) found that heritable TBAD was the cause of TBAD in one of four patients, and familial TBAD was presented at an early age. Finally, it has been established that hypertension is an essential component of both familial TBAD and sporadic TBAD (Shalhub et al., 2020).

Most patients in our TBAD cohort were male, most had hypertension, and few of them had diabetes. These population characteristics are consistent with the reported TBAD in the Chinese population (Huang et al., 2021). Based on whole genome sequencing data, we demonstrated that the *ULK4* gene is associated with the age of TBAD onset based on both the SKAT and 2-sample MR analyses. Furthermore, we found that DNA methylation of cg25209153_ULK4_ and expression of *ULK4* were associated with the age of first onset. Furthermore, high DNA methylation of cg25209153_ULK4_ was negatively correlated with age of first onset. Inversely, high expression of *ULK4* was positively correlated with age of first onset.

Several GWASs have reported significant associations of *ULK4* SNPs with hypertension in individuals of European (particularly those with high diastolic blood pressure), African American, and East Asian descent (Guo et al., 2016). It is also known that poorly controlled hypertension is a major risk factor for TBAD, and our study demonstrated that the *ULK4* gene, involved in endocytosis and axon growth (Ostberg et al., 2020), is associated with age of first onset of TBAD, suggesting that in addition to the association with the control of hypertension, genomic variants in *ULK4* have a potential mechanism for contributing to the early onset of TBAD. As autophagy is a highly conserved catabolic process and a major cellular pathway for the degradation of long-lived proteins and cytoplasmic organelles, *ULK4* plays critical roles in autophagy and dysregulation of autophagy may lead to the early onset of TBAD. Further studies are needed to validate the link between *ULK4* and the age of first onset of TBAD. *ULK4* may be an effective companion diagnostic target in TBAD if it is confirmed by further fundamental and clinical studies.

In addition, our study will follow up the prognosis of the existing cases so as to suggest early detection and early treatments for at-risk patients. At the same time, we will also consider measuring different omics data such as proteomics from the same batch to further validate the importance of the ULK4 gene in TBAD.

## Conclusion and limitations

Our study also has some limitations. First, the sample size was limited, which may impact the robustness of the 2-sample MR analyses. Second, because only TBAD patients were included in our study, the association of *ULK4* with type A dissections could not be investigated; moreover, as a case-only study, which did not involve the control group, the current study cannot conclude the effective companion diagnostic target in TBAD. Third, the validated DNA samples were not collected. In addition, the clinical information was not comprehensive, such as concomitant medication of antiplatelet and statin, the potential bias risk may impact the robustness of the results.

In conclusion, this is the first study of the *ULK* gene family in TBAD to focus on an analysis of the association with the first onset age. We demonstrated that the *ULK4* gene is associated with the age of first onset of TBAD based on both the SKAT and 2-sample MR analyses. *ULK4* may be an effective companion diagnostic target in TBAD.

## Data Availability

The data presented in the study are deposited through these following websites. The eQTL summary data can be found in http://www.psychENCODE.org/. The mQTL data from the meta-analyses of Brisbane Systems Genetics Study and the Lothian Birth Cohorts of 1921 and 1936 are available at http://cnsgenomics.com/software/smr/#Download. The whole genome sequencing data of TBAD cohort was provided by Yi Si, et al. Requests to access this dataset should be directed to sysiy@yahoo.com.

## Abbreviations

eQTL, expression quantitative trait loci; KAT, sequence kernel association test; MAF, minor allele frequency; MFS, Marfan syndrome; mQTL, methylation quantitative trait loci; MR, Mendelian randomization; STBAD, type B aortic dissection; VSMCs, vascular smooth muscle cells.
