# Supplementary material for: Association of genetic variants in ULK4 with the age of first onset of type B aortic dissection
Source: Front Genet. 2022 Sep 2;13:956866. doi: 10.3389/fgene.2022.956866 (PMC9478570; doi:10.3389/fgene.2022.956866)
Supplement: Supplementary file 1 [file DataSheet1.docx]

**Supplement**

This supplement has been provided by the authors to give readers additional information about their works

Supplement to: Lihong Huang, Jiaqi Tang, Lijuan Lin, Ruihan Wang, Feng Chen, Yongyue Wei, Yi Si, Weiguo Fu. ***Association of genetic variants in ULK4 with the age of first onset of type B aortic dissection***.

**Table S1: Demographic and clinical descriptions for type B aortic dissection patients**

| **Characteristics** |  | **AD cohort**  **(N=159)** |
| --- | --- | --- |
| Age, mean±SD（min, max） |  | 56.11±14.33 (18, 85) |
| Gender, n(%) |  |  |
|  | Female | 34 (21.38) |
|  | Male | 125 (78.62) |
| First onset age, mean±SD |  | 54.89±14.51 |
| Complex dissection, n(%) |  |  |
|  | Complex | 102 (64.15) |
|  | Non-complex | 57 (35.85) |
| Smoking status, n(%) |  |  |
|  | Never | 3 (1.89) |
|  | Former | 94 (59.12) |
|  | Current | 62 (38.99) |
| Diabetes, n(%) |  |  |
|  | Yes | 16 (10.06) |
|  | No | 143 (89.94) |
| Hypertension, n(%) |  |  |
|  | Yes | 141 (88.68) |
|  | No | 18 (11.32) |
| Hypertension classification, n(%) |  |  |
|  | No hypertension history | 15 (9.43) |
|  | I | 110 (69.18) |
|  | II | 16 (10.06) |
|  | III | 16 (10.06) |
|  | Missing | 2 (1.26) |
| Hypertension controlling, n(%) |  |  |
|  | No controlling | 18 (11.32) |
|  | Good controlling | 103 (64.78) |
|  | Poor controlling | 38 (23.90) |
| Renal function, n(%) |  |  |
|  | Normal | 68 (42.77) |
|  | Phase II | 66 (41.51) |
|  | Phase III | 25 (15.72) |
| Cerebrovascular assessment, n(%) |  |  |
|  | No cerebral infarction | 151 (94.97) |
|  | TIA or cerebellar infarction | 8 (5.03) |
| Cardiac assessment, n(%) |  |  |
|  | Normal | 117 (73.58) |
|  | PCI postoperative | 10 (6.28) |
|  | Arrhythmology | 8 (5.03) |
|  | Cardiac dysfunction | 21 (13.21) |
|  | Pericardial effusion | 3 (1.89) |
| Lung condition, n(%) |  |  |
|  | Normal | 118 (74.21) |
|  | Pleural effusion | 18 (11.32) |
|  | Pneumonia | 13 (8.18) |
|  | COPD | 10 (6.29) |

Sum of frequency numbers may not equal to the total sample size due to missing values.

Data is presented as mean ± SD for continuous variables and n (%) for categorical variables.

Coefficients and p values represent results of linear regression for continuous variables and categorical variables.

Data source: TBAD cohort.

**Table S2: Association results of late-onset analysis for the snp sites of ULK4**

| **SNP** | **Chr** | **Position** | **Allele** | **MAF** | **Coefficient (95%Cl)** | ***P*** | **q-FDR** |
| --- | --- | --- | --- | --- | --- | --- | --- |
| rs191792955 | 3 | 41529173 | C>A | 0.0314 | -17.64 (-26.254,-9.034) | <0.0001 | 0.0031 |
| rs116932804 | 3 | 41538466 | G>C | 0.0314 | -17.64 (-26.254,-9.034) | <0.0001 | 0.0031 |
| rs78358855 | 3 | 41548392 | A>G | 0.0314 | -17.64 (-26.254,-9.034) | <0.0001 | 0.0031 |
| rs117464550 | 3 | 41564761 | A>G | 0.0314 | -17.64 (-26.254,-9.034) | <0.0001 | 0.0031 |
| rs73830221 | 3 | 41577765 | T>A | 0.0314 | -17.64 (-26.254,-9.034) | <0.0001 | 0.0031 |
| rs141669418 | 3 | 41595405 | G>A | 0.0314 | -17.64 (-26.254,-9.034) | <0.0001 | 0.0031 |
| rs7613306 | 3 | 41601832 | T>C | 0.0314 | -17.64 (-26.254,-9.034) | <0.0001 | 0.0031 |
| rs7631990 | 3 | 41608132 | A>T | 0.0314 | -17.64 (-26.254,-9.034) | <0.0001 | 0.0031 |
| rs56289557 | 3 | 41590966 | T>G | 0.0346 | -15.36 (-22.879,-7.844) | <0.0001 | 0.0031 |
| rs58006056 | 3 | 41585684 | C>T | 0.0319 | -17.56 (-26.197,-8.933) | <0.0001 | 0.0031 |
| rs138574068 | 3 | 41817680 | T>C | 0.0377 | -14.86 (-22.917,-6.811) | 0.0004 | 0.0111 |
| rs142574024 | 3 | 41416756 | G>C | 0.0283 | -16.31 (-25.534,-7.088) | 0.0006 | 0.0134 |
| rs116552297 | 3 | 41426835 | G>A | 0.0283 | -16.31 (-25.534,-7.088) | 0.0006 | 0.0134 |
| rs1586454 | 3 | 41450568 | G>A | 0.0283 | -16.31 (-25.534,-7.088) | 0.0006 | 0.0134 |
| rs11921851 | 3 | 41497632 | C>A | 0.0283 | -16.31 (-25.534,-7.088) | 0.0006 | 0.0134 |
| rs74282513 | 3 | 41703190 | T>C | 0.1824 | -6.76 (-10.754,-2.773) | 0.0010 | 0.0207 |
| rs56191895 | 3 | 41390319 | C>T | 0.0912 | -8.10 (-13.310,-2.886) | 0.0026 | 0.0484 |
| rs17058895 | 3 | 41615845 | T>G | 0.1226 | -7.95 (-13.136,-2.763) | 0.0029 | 0.0524 |
| rs77473848 | 3 | 41749357 | G>A | 0.239 | 5.67 (1.698,9.649) | 0.0055 | 0.0896 |
| rs12633534 | 3 | 41789150 | T>C | 0.3176 | 4.89 (1.392,8.384) | 0.0065 | 0.1013 |
| rs74763606 | 3 | 41918607 | A>G | 0.022 | 12.55 (2.944,22.164) | 0.0108 | 0.1592 |
| rs73073283 | 3 | 42027018 | G>C | 0.3019 | 4.53 (1.052,8.006) | 0.0111 | 0.1592 |
| rs2888057 | 3 | 41441627 | G>A | 0.066 | -8.28 (-14.713,-1.848) | 0.0120 | 0.1663 |
| rs17062195 | 3 | 41811891 | G>A | 0.0314 | -11.55 (-20.606,-2.503) | 0.0127 | 0.1697 |
| rs3733048 | 3 | 41900593 | T>C | 0.0252 | 11.40 (2.327,20.475) | 0.0142 | 0.1821 |
| rs62257301 | 3 | 42048851 | G>A | 0.0506 | -7.89 (-14.703,-1.069) | 0.0237 | 0.2833 |
| rs2683694 | 3 | 41915374 | T>G | 0.2722 | -4.12 (-7.740,-0.503) | 0.0259 | 0.2914 |
| rs117670723 | 3 | 41292002 | G>C | 0.0314 | -10.39 (-19.641,-1.144) | 0.0279 | 0.3045 |
| rs900569 | 3 | 41859973 | C>T | 0.4497 | -3.71 (-7.079,-0.345) | 0.0310 | 0.3087 |
| rs141039673 | 3 | 41515088 | G>A | 0.0126 | 15.50 (1.304,29.702) | 0.0326 | 0.3087 |
| rs546220193 | 3 | 41577470 | G>A | 0.0126 | 15.50 (1.304,29.702) | 0.0326 | 0.3087 |
| rs9822227 | 3 | 41468890 | T>A | 0.1195 | -5.42 (-10.540,-0.304) | 0.0380 | 0.3423 |
| rs1717022 | 3 | 41984041 | A>G | 0.4937 | -3.42 (-6.751,-0.084) | 0.0446 | 0.3915 |
| rs35391137 | 3 | 41617623 | A>G | 0.0535 | -6.73 (-13.376,-0.087) | 0.0471 | 0.3951 |
| rs11720271 | 3 | 41852434 | A>G | 0.0377 | -8.39 (-16.793,0.015) | 0.0504 | 0.3951 |
| rs62258621 | 3 | 41867474 | A>G | 0.0377 | -8.39 (-16.793,0.015) | 0.0504 | 0.3951 |
| rs11719319 | 3 | 41900524 | T>C | 0.0377 | -8.39 (-16.793,0.015) | 0.0504 | 0.3951 |
| rs192845249 | 3 | 41320492 | G>A | 0.0126 | 13.97 (-0.030,27.972) | 0.0505 | 0.3951 |
| rs1716979 | 3 | 41961168 | C>T | 0.4591 | -3.23 (-6.545,0.080) | 0.0557 | 0.4136 |
| rs190974819 | 3 | 41763039 | T>C | 0.0157 | 10.52 (-0.377,21.410) | 0.0584 | 0.4136 |
| rs117195185 | 3 | 41780574 | T>C | 0.0252 | -10.45 (-21.283,0.381) | 0.0585 | 0.4136 |
| rs9860278 | 3 | 41543301 | A>G | 0.1132 | -5.22 (-10.737,0.290) | 0.0631 | 0.4136 |
| rs9841327 | 3 | 41557382 | G>A | 0.1132 | -5.22 (-10.737,0.290) | 0.0631 | 0.4136 |
| rs4580521 | 3 | 41576183 | A>C | 0.1132 | -5.22 (-10.737,0.290) | 0.0631 | 0.4136 |
| rs117618946 | 3 | 41437139 | A>G | 0.0346 | -9.21 (-19.006,0.578) | 0.0649 | 0.4136 |
| rs79972182 | 3 | 41456904 | C>T | 0.0346 | -9.21 (-19.006,0.578) | 0.0649 | 0.4136 |
| rs138200882 | 3 | 41737545 | G>A | 0.022 | -10.99 (-22.788,0.814) | 0.0678 | 0.4136 |
| rs75963284 | 3 | 41855288 | C>A | 0.022 | -10.99 (-22.788,0.814) | 0.0678 | 0.4136 |
| rs62257277 | 3 | 42004228 | C>T | 0.022 | -10.99 (-22.788,0.814) | 0.0678 | 0.4136 |
| rs182850014 | 3 | 42038093 | C>G | 0.022 | -10.99 (-22.788,0.814) | 0.0678 | 0.4136 |
| rs139358516 | 3 | 41475983 | A>G | 0.0157 | 11.30 (-1.377,23.983) | 0.0802 | 0.4770 |
| rs76301801 | 3 | 41987079 | G>A | 0.0409 | -7.17 (-15.223,0.890) | 0.0808 | 0.4770 |
| rs77838233 | 3 | 41834371 | T>C | 0.0409 | -7.53 (-16.084,1.019) | 0.0838 | 0.4845 |
| rs143506729 | 3 | 41903961 | A>G | 0.022 | 9.35 (-1.579,20.274) | 0.0930 | 0.5177 |
| rs184581802 | 3 | 41410213 | T>C | 0.0252 | -9.43 (-20.471,1.609) | 0.0935 | 0.5177 |
| rs4973952 | 3 | 41525357 | G>C | 0.1289 | -4.15 (-9.140,0.833) | 0.1018 | 0.5553 |
| rs10510726 | 3 | 41724117 | A>G | 0.1604 | -3.53 (-7.806,0.739) | 0.1043 | 0.5603 |
| rs145931274 | 3 | 41485186 | T>C | 0.0191 | 8.50 (-1.933,18.941) | 0.1094 | 0.5710 |
| rs2371488 | 3 | 41313475 | G>T | 0.3774 | -2.50 (-5.634,0.634) | 0.1170 | 0.5729 |
| rs76115364 | 3 | 41589393 | G>A | 0.0283 | 7.95 (-2.037,17.934) | 0.1178 | 0.5729 |
| rs151247442 | 3 | 41582103 | C>A | 0.0346 | 7.13 (-1.838,16.089) | 0.1183 | 0.5729 |
| rs1390341 | 3 | 41606936 | C>T | 0.0346 | 7.13 (-1.838,16.089) | 0.1183 | 0.5729 |
| rs146184122 | 3 | 41975370 | C>T | 0.0189 | -10.17 (-22.990,2.659) | 0.1193 | 0.5729 |
| rs80126279 | 3 | 41828699 | G>C | 0.022 | -8.65 (-19.687,2.393) | 0.1237 | 0.5780 |
| rs76745877 | 3 | 41811937 | T>C | 0.022 | -9.22 (-21.030,2.586) | 0.1248 | 0.5780 |
| rs4973981 | 3 | 41693047 | A>G | 0.1698 | -3.27 (-7.460,0.921) | 0.1252 | 0.5780 |
| rs9821358 | 3 | 41583176 | G>A | 0.1101 | -4.31 (-9.912,1.297) | 0.1309 | 0.5931 |
| rs147882461 | 3 | 41592953 | C>T | 0.0252 | 7.74 (-2.355,17.833) | 0.1318 | 0.5931 |
| rs62258656 | 3 | 41944757 | A>G | 0.0409 | -6.21 (-14.385,1.965) | 0.1354 | 0.6018 |
| rs9311266 | 3 | 41315914 | T>G | 0.0786 | -4.21 (-9.831,1.404) | 0.1404 | 0.6163 |
| rs146226439 | 3 | 42009847 | T>C | 0.0252 | 7.58 (-2.669,17.838) | 0.1459 | 0.6327 |
| rs9878048 | 3 | 41351287 | A>G | 0.3758 | -2.30 (-5.430,0.832) | 0.1488 | 0.6372 |
| rs138865096 | 3 | 41308005 | C>T | 0.0157 | 9.40 (-3.452,22.248) | 0.1504 | 0.6372 |
| rs72864992 | 3 | 41396066 | T>A | 0.0535 | 4.94 (-1.904,11.789) | 0.1558 | 0.6460 |
| rs145768774 | 3 | 41290900 | G>A | 0.0157 | 9.09 (-3.512,21.686) | 0.1561 | 0.6460 |
| rs13315196 | 3 | 41399334 | A>C | 0.0535 | -5.15 (-12.379,2.077) | 0.1610 | 0.6557 |
| rs4973930 | 3 | 41306063 | C>A | 0.4874 | 2.47 (-1.004,5.939) | 0.1621 | 0.6557 |
| rs182760397 | 3 | 41542615 | A>C | 0.0252 | 6.30 (-2.683,15.289) | 0.1677 | 0.6637 |
| rs13084436 | 3 | 42017537 | A>T | 0.2138 | -2.71 (-6.568,1.153) | 0.1678 | 0.6637 |
| rs59142862 | 3 | 41409836 | G>A | 0.0566 | -4.89 (-11.926,2.146) | 0.1716 | 0.6716 |
| rs75057445 | 3 | 41635390 | G>C | 0.0943 | -3.69 (-9.059,1.680) | 0.1765 | 0.6758 |
| rs61745795 | 3 | 41759288 | T>C | 0.0314 | 6.15 (-2.988,15.280) | 0.1856 | 0.6933 |
| rs78719651 | 3 | 41926869 | T>G | 0.0314 | 6.15 (-2.988,15.280) | 0.1856 | 0.6933 |
| rs140593441 | 3 | 41610868 | A>C | 0.0157 | 8.59 (-4.218,21.405) | 0.1870 | 0.6933 |
| rs7616123 | 3 | 41798941 | A>G | 0.2138 | -2.65 (-6.690,1.387) | 0.1965 | 0.7144 |
| rs9810731 | 3 | 41882101 | C>T | 0.2083 | -2.62 (-6.655,1.422) | 0.2023 | 0.7212 |
| rs190157839 | 3 | 41683400 | C>T | 0.0346 | 5.48 (-3.085,14.041) | 0.2080 | 0.7342 |
| rs10461009 | 3 | 41344582 | C>A | 0.0755 | -3.59 (-9.277,2.095) | 0.2139 | 0.7403 |
| rs1487569 | 3 | 41368428 | C>T | 0.0755 | -3.59 (-9.277,2.095) | 0.2139 | 0.7403 |
| rs370268048 | 3 | 41600363 | A>G | 0.0221 | 5.86 (-3.575,15.293) | 0.2216 | 0.7441 |
| rs146294066 | 3 | 41510090 | T>C | 0.0283 | -6.07 (-15.908,3.761) | 0.2241 | 0.7441 |
| rs117494100 | 3 | 41467951 | G>A | 0.0409 | 5.29 (-3.293,13.871) | 0.2251 | 0.7441 |
| rs199743067 | 3 | 41414672 | A>G | 0.0126 | 9.55 (-6.040,25.138) | 0.2279 | 0.7441 |
| rs6763756 | 3 | 41369049 | C>T | 0.3805 | -1.87 (-4.955,1.216) | 0.2330 | 0.7441 |
| rs145908990 | 3 | 41597132 | C>G | 0.0126 | -8.62 (-22.887,5.638) | 0.2339 | 0.7441 |
| rs117104880 | 3 | 41666919 | T>C | 0.0126 | -8.62 (-22.887,5.638) | 0.2339 | 0.7441 |
| rs138610411 | 3 | 41437928 | T>G | 0.0314 | 5.18 (-3.421,13.788) | 0.2357 | 0.7441 |
| rs76883444 | 3 | 41499924 | T>C | 0.0126 | -8.59 (-22.919,5.746) | 0.2382 | 0.7441 |
| rs571926141 | 3 | 41408917 | G>A | 0.0126 | -8.53 (-23.029,5.970) | 0.2468 | 0.7441 |
| rs542037418 | 3 | 41455532 | C>T | 0.0126 | -8.53 (-23.029,5.970) | 0.2468 | 0.7441 |
| rs190149684 | 3 | 41490636 | T>C | 0.0126 | -8.53 (-23.029,5.970) | 0.2468 | 0.7441 |
| rs2700464 | 3 | 41522811 | C>T | 0.066 | 3.96 (-2.785,10.699) | 0.2479 | 0.7441 |
| rs117137091 | 3 | 41333671 | A>G | 0.0283 | 5.12 (-3.659,13.907) | 0.2507 | 0.7441 |
| rs964351 | 3 | 41432472 | C>T | 0.4874 | 1.74 (-1.249,4.730) | 0.2517 | 0.7441 |
| rs1795346 | 3 | 41609466 | G>C | 0.4969 | -1.90 (-5.240,1.446) | 0.2637 | 0.7719 |
| rs184765208 | 3 | 42035104 | A>G | 0.0126 | 8.01 (-6.298,22.322) | 0.2702 | 0.7828 |
| rs77877710 | 3 | 41985608 | C>T | 0.0535 | -4.15 (-11.581,3.285) | 0.2718 | 0.7828 |
| rs1386600 | 3 | 41448287 | T>C | 0.4969 | 1.63 (-1.334,4.602) | 0.2782 | 0.7901 |
| rs75620919 | 3 | 42015832 | T>G | 0.0472 | -4.38 (-12.358,3.595) | 0.2793 | 0.7901 |
| rs9816636 | 3 | 41386530 | C>T | 0.3931 | -1.69 (-4.776,1.399) | 0.2813 | 0.7901 |
| rs55763747 | 3 | 41409762 | A>G | 0.3868 | -1.67 (-4.747,1.402) | 0.2841 | 0.7901 |
| rs950340005 | 3 | 41748377 | G>A | 0.0157 | -7.14 (-20.302,6.021) | 0.2853 | 0.7901 |
| rs184229083 | 3 | 41483459 | T>C | 0.0252 | 4.72 (-4.250,13.681) | 0.3002 | 0.8125 |
| rs78154651 | 3 | 41516366 | C>T | 0.0252 | 4.72 (-4.250,13.681) | 0.3002 | 0.8125 |
| rs76888198 | 3 | 41883678 | A>T | 0.1101 | 2.68 (-2.545,7.896) | 0.3126 | 0.8142 |
| rs74927080 | 3 | 41843989 | C>T | 0.0881 | 3.06 (-2.946,9.061) | 0.3157 | 0.8142 |
| rs147349601 | 3 | 41712068 | A>T | 0.0414 | -4.44 (-13.157,4.281) | 0.3159 | 0.8142 |
| rs1495699 | 3 | 41532752 | A>G | 0.1321 | -2.36 (-7.001,2.283) | 0.3168 | 0.8142 |
| rs148964615 | 3 | 41683253 | G>A | 0.0126 | 7.06 (-7.104,21.219) | 0.3262 | 0.8142 |
| rs116888971 | 3 | 41788940 | G>C | 0.0126 | 7.06 (-7.104,21.219) | 0.3262 | 0.8142 |
| rs139045537 | 3 | 41883960 | T>C | 0.0126 | 7.06 (-7.104,21.219) | 0.3262 | 0.8142 |
| rs150692193 | 3 | 41946069 | G>A | 0.0126 | 7.06 (-7.104,21.219) | 0.3262 | 0.8142 |
| rs144687328 | 3 | 42016162 | G>C | 0.0126 | 7.06 (-7.104,21.219) | 0.3262 | 0.8142 |
| rs4973982 | 3 | 41710175 | G>C | 0.022 | 5.33 (-5.513,16.17) | 0.3328 | 0.8142 |
| rs35908999 | 3 | 41725319 | T>C | 0.022 | 5.33 (-5.513,16.17) | 0.3328 | 0.8142 |
| rs148054772 | 3 | 41727305 | C>T | 0.0252 | 5.12 (-5.334,15.566) | 0.3348 | 0.8142 |
| rs118118483 | 3 | 41811949 | T>C | 0.0409 | 3.98 (-4.156,12.114) | 0.3352 | 0.8142 |
| rs6763508 | 3 | 41750989 | T>C | 0.2107 | -1.98 (-6.045,2.080) | 0.3363 | 0.8142 |
| rs117017364 | 3 | 41680203 | C>G | 0.0283 | 4.73 (-4.990,14.450) | 0.3377 | 0.8142 |
| rs145760861 | 3 | 41346350 | C>T | 0.0189 | 5.73 (-6.080,17.549) | 0.3389 | 0.8142 |
| rs374933475 | 3 | 41345291 | G>A | 0.0126 | 7.05 (-7.532,21.633) | 0.3408 | 0.8142 |
| rs193059734 | 3 | 41666103 | C>G | 0.0189 | 4.79 (-5.147,14.717) | 0.3425 | 0.8142 |
| rs117650857 | 3 | 41902869 | A>G | 0.0314 | -4.51 (-13.896,4.876) | 0.3438 | 0.8142 |
| rs77864909 | 3 | 41333679 | T>A | 0.0409 | 4.20 (-4.622,13.017) | 0.3484 | 0.8188 |
| rs117534481 | 3 | 41391221 | T>C | 0.0189 | -5.69 (-17.700,6.316) | 0.3503 | 0.8188 |
| rs550401990 | 3 | 41419847 | C>G | 0.0126 | -6.79 (-21.273,7.697) | 0.3558 | 0.8255 |
| rs34636420 | 3 | 41746085 | T>C | 0.0126 | 6.5 (-7.621,20.616) | 0.3645 | 0.8255 |
| rs7642694 | 3 | 41646067 | T>C | 0.1855 | -1.91 (-6.072,2.253) | 0.3660 | 0.8255 |
| rs183457880 | 3 | 41512252 | G>A | 0.022 | 5.37 (-6.385,17.132) | 0.3678 | 0.8255 |
| rs9852891 | 3 | 41398900 | C>T | 0.3868 | -1.40 (-4.475,1.669) | 0.3681 | 0.8255 |
| rs149823439 | 3 | 41817299 | A>G | 0.0252 | -4.65 (-14.967,5.674) | 0.3749 | 0.8255 |
| rs562280032 | 3 | 41644140 | C>T | 0.0189 | -5.24 (-16.948,6.476) | 0.3783 | 0.8255 |
| rs113199954 | 3 | 41850653 | G>A | 0.0377 | 3.75 (-4.654,12.157) | 0.3791 | 0.8255 |
| rs74558506 | 3 | 41583175 | C>T | 0.0189 | -5.26 (-17.070,6.553) | 0.3803 | 0.8255 |
| rs141097530 | 3 | 41605044 | G>A | 0.0189 | -5.26 (-17.070,6.553) | 0.3803 | 0.8255 |
| rs139748223 | 3 | 41660090 | T>G | 0.0189 | -5.26 (-17.070,6.553) | 0.3803 | 0.8255 |
| rs117603998 | 3 | 41520927 | C>T | 0.0377 | -4.00 (-13.001,5.004) | 0.3814 | 0.8255 |
| rs951161024 | 3 | 41951371 | T>C | 0.0126 | 6.40 (-8.112,20.916) | 0.3847 | 0.8255 |
| rs6809441 | 3 | 41494605 | T>C | 0.1195 | -2.47 (-8.084,3.139) | 0.3852 | 0.8255 |
| rs1273205 | 3 | 41619894 | T>G | 0.4686 | 1.42 (-1.840,4.686) | 0.3900 | 0.8308 |
| rs116985872 | 3 | 41780787 | T>C | 0.022 | -4.65 (-15.537,6.243) | 0.4003 | 0.8421 |
| rs138577669 | 3 | 42036947 | T>C | 0.022 | -4.65 (-15.537,6.243) | 0.4003 | 0.8421 |
| rs7613809 | 3 | 41355694 | T>C | 0.0786 | -2.31 (-7.741,3.124) | 0.4023 | 0.8421 |
| rs150345968 | 3 | 41328676 | G>A | 0.0157 | -5.41 (-18.316,7.492) | 0.4084 | 0.8435 |
| rs139555497 | 3 | 41747013 | C>T | 0.0126 | -5.81 (-20.009,8.386) | 0.4197 | 0.8435 |
| rs142994657 | 3 | 41306144 | G>A | 0.0126 | -5.78 (-20.000,8.434) | 0.4226 | 0.8435 |
| rs181096053 | 3 | 41810629 | T>A | 0.0126 | 5.71 (-8.406,19.827) | 0.4252 | 0.8435 |
| rs60659132 | 3 | 41678906 | C>T | 0.3774 | 1.39 (-2.085,4.868) | 0.4302 | 0.8435 |
| rs1256353 | 3 | 41628218 | T>C | 0.1101 | 2.11 (-3.206,7.427) | 0.4338 | 0.8435 |
| rs75008165 | 3 | 41496863 | A>C | 0.044 | 3.16 (-5.016,11.341) | 0.4459 | 0.8435 |
| rs117548965 | 3 | 41745417 | G>C | 0.0503 | 2.86 (-4.549,10.261) | 0.4471 | 0.8435 |
| rs77212658 | 3 | 41601916 | T>C | 0.0912 | -2.30 (-8.265,3.669) | 0.4477 | 0.8435 |
| rs7620446 | 3 | 41455776 | G>A | 0.1069 | -2.02 (-7.289,3.240) | 0.4484 | 0.8435 |
| rs994439 | 3 | 41716317 | A>G | 0.3176 | -1.31 (-4.760,2.140) | 0.4539 | 0.8435 |
| rs146184008 | 3 | 41588866 | G>A | 0.0535 | 2.74 (-4.473,9.947) | 0.4543 | 0.8435 |
| rs139911690 | 3 | 41660743 | A>G | 0.0346 | 3.50 (-5.720,12.717) | 0.4543 | 0.8435 |
| rs76191830 | 3 | 41981867 | C>A | 0.0189 | -4.50 (-16.380,7.374) | 0.4548 | 0.8435 |
| rs34607988 | 3 | 42023735 | T>A | 0.0189 | -4.50 (-16.380,7.374) | 0.4548 | 0.8435 |
| rs3774377 | 3 | 41938522 | T>A | 0.0535 | 2.73 (-4.483,9.938) | 0.4558 | 0.8435 |
| rs190172159 | 3 | 41952970 | T>C | 0.0157 | -4.80 (-17.519,7.910) | 0.4563 | 0.8435 |
| rs191561886 | 3 | 41604274 | C>T | 0.0283 | 3.86 (-6.381,14.092) | 0.4577 | 0.8435 |
| rs75133830 | 3 | 41997749 | T>C | 0.0503 | 2.75 (-4.653,10.158) | 0.4636 | 0.8435 |
| rs79635927 | 3 | 41590720 | G>A | 0.0157 | 5.16 (-8.749,19.074) | 0.4644 | 0.8435 |
| rs58537812 | 3 | 41664944 | C>T | 0.0346 | 3.32 (-5.660,12.291) | 0.4664 | 0.8435 |
| rs149124189 | 3 | 41341298 | C>T | 0.0346 | 3.23 (-5.555,12.009) | 0.4688 | 0.8435 |
| rs150423127 | 3 | 41494403 | T>C | 0.0252 | 3.98 (-6.873,14.833) | 0.4697 | 0.8435 |
| rs1586453 | 3 | 41450625 | T>C | 0.1226 | 1.81 (-3.148,6.762) | 0.4721 | 0.8435 |
| rs35766766 | 3 | 41693765 | T>G | 0.0597 | -2.43 (-9.108,4.251) | 0.4735 | 0.8435 |
| rs117368452 | 3 | 41579809 | T>G | 0.0566 | 2.55 (-4.496,9.594) | 0.4756 | 0.8435 |
| rs118055478 | 3 | 41425452 | A>C | 0.0126 | 5.45 (-9.871,20.766) | 0.4832 | 0.8471 |
| rs6599158 | 3 | 41545321 | A>G | 0.4652 | -1.21 (-4.627,2.207) | 0.4850 | 0.8471 |
| rs12152316 | 3 | 41386200 | T>C | 0.0503 | 2.47 (-4.537,9.48) | 0.4869 | 0.8471 |
| rs149674212 | 3 | 41447095 | T>A | 0.0314 | 3.08 (-5.668,11.837) | 0.4871 | 0.8471 |
| rs79857952 | 3 | 41998080 | C>T | 0.0629 | -2.24 (-8.789,4.314) | 0.5007 | 0.8648 |
| rs147743799 | 3 | 41873220 | T>C | 0.0157 | 4.42 (-8.567,17.409) | 0.5020 | 0.8648 |
| rs118043132 | 3 | 41740993 | T>G | 0.0189 | 3.93 (-7.737,15.599) | 0.5064 | 0.8682 |
| rs4544584 | 3 | 41406328 | G>T | 0.0472 | 2.39 (-4.832,9.621) | 0.5135 | 0.8748 |
| rs80278777 | 3 | 41496740 | G>A | 0.044 | 2.35 (-4.907,9.600) | 0.5235 | 0.8750 |
| rs77840604 | 3 | 41586065 | A>G | 0.0377 | 2.93 (-6.158,12.016) | 0.5250 | 0.8750 |
| rs78783786 | 3 | 41616667 | A>C | 0.0377 | 2.93 (-6.158,12.016) | 0.5250 | 0.8750 |
| rs189431555 | 3 | 41923996 | A>G | 0.0126 | -5.18 (-21.48,11.125) | 0.5311 | 0.8771 |
| rs9816360 | 3 | 41416476 | C>T | 0.0446 | 2.17 (-5.273,9.615) | 0.5651 | 0.9185 |
| rs184530687 | 3 | 41498410 | G>A | 0.0126 | 4.14 (-10.256,18.527) | 0.5709 | 0.9185 |
| rs80029789 | 3 | 41727988 | G>T | 0.022 | -3.13 (-14.055,7.799) | 0.5723 | 0.9185 |
| rs117048196 | 3 | 41780204 | C>A | 0.0189 | -3.34 (-15.251,8.561) | 0.5795 | 0.9185 |
| rs181655439 | 3 | 41821976 | C>T | 0.0189 | -3.34 (-15.251,8.561) | 0.5795 | 0.9185 |
| rs184213561 | 3 | 41851070 | G>A | 0.0189 | -3.34 (-15.251,8.561) | 0.5795 | 0.9185 |
| rs77881285 | 3 | 41507149 | C>T | 0.4874 | -1.01 (-4.639,2.623) | 0.5840 | 0.9185 |
| rs186456241 | 3 | 41490331 | T>C | 0.0126 | -3.95 (-18.187,10.289) | 0.5843 | 0.9185 |
| rs3897976 | 3 | 41606692 | G>A | 0.0786 | 1.64 (-4.31,7.588) | 0.5869 | 0.9185 |
| rs34306571 | 3 | 41420378 | G>A | 0.0629 | 1.88 (-5.016,8.766) | 0.5915 | 0.9185 |
| rs6786286 | 3 | 41377659 | A>G | 0.0597 | -1.89 (-8.854,5.066) | 0.5915 | 0.9185 |
| rs9852315 | 3 | 41340824 | A>G | 0.0566 | -1.90 (-8.957,5.163) | 0.5961 | 0.9185 |
| rs1716699 | 3 | 41898272 | C>T | 0.2075 | 1.06 (-2.927,5.047) | 0.5999 | 0.9185 |
| rs9869446 | 3 | 41851210 | G>A | 0.2044 | 1.07 (-2.951,5.082) | 0.6008 | 0.9185 |
| rs142164660 | 3 | 41311128 | A>G | 0.022 | 2.86 (-7.958,13.688) | 0.6016 | 0.9185 |
| rs144640939 | 3 | 41759125 | C>G | 0.022 | 2.80 (-8.226,13.824) | 0.6165 | 0.9281 |
| rs148443395 | 3 | 41383459 | T>C | 0.0157 | -3.20 (-15.839,9.448) | 0.6181 | 0.9281 |
| rs145687790 | 3 | 41996332 | G>A | 0.022 | 2.55 (-7.589,12.689) | 0.6198 | 0.9281 |
| rs117498645 | 3 | 41666959 | G>A | 0.0189 | 2.94 (-9.034,14.904) | 0.6286 | 0.9281 |
| rs75957696 | 3 | 41701211 | T>G | 0.0189 | 2.94 (-9.034,14.904) | 0.6286 | 0.9281 |
| rs34575078 | 3 | 41741571 | T>C | 0.0472 | 1.69 (-5.235,8.621) | 0.6298 | 0.9281 |
| rs143235387 | 3 | 42038715 | T>G | 0.0157 | -3.06 (-15.626,9.506) | 0.6309 | 0.9281 |
| rs12330747 | 3 | 41990829 | C>A | 0.239 | -0.99 (-5.043,3.071) | 0.6316 | 0.9281 |
| rs79351593 | 3 | 41799417 | C>T | 0.0692 | 1.57 (-4.950,8.092) | 0.6346 | 0.9287 |
| rs148185098 | 3 | 41316721 | G>C | 0.0157 | 2.96 (-9.926,15.843) | 0.6506 | 0.9295 |
| rs187227556 | 3 | 41352600 | G>A | 0.0157 | 2.96 (-9.926,15.843) | 0.6506 | 0.9295 |
| rs184317932 | 3 | 41754539 | G>A | 0.0189 | 2.42 (-8.107,12.939) | 0.6506 | 0.9295 |
| rs180860375 | 3 | 41465851 | G>A | 0.0314 | -2.09 (-11.270,7.096) | 0.6539 | 0.9295 |
| rs542313730 | 3 | 42012509 | C>T | 0.0157 | 2.87 (-9.875,15.609) | 0.6571 | 0.9295 |
| rs139057755 | 3 | 41449513 | T>C | 0.0314 | -2.00 (-11.150,7.160) | 0.6672 | 0.9295 |
| rs75571497 | 3 | 41861165 | A>G | 0.0629 | 1.43 (-5.221,8.088) | 0.6708 | 0.9295 |
| rs7629070 | 3 | 41440406 | C>T | 0.0723 | -1.31 (-7.504,4.880) | 0.6760 | 0.9295 |
| rs180868872 | 3 | 41898121 | A>G | 0.0127 | 3.07 (-11.410,17.543) | 0.6760 | 0.9295 |
| rs149661935 | 3 | 41358132 | T>C | 0.0157 | 2.18 (-8.441,12.801) | 0.6855 | 0.9295 |
| rs187731418 | 3 | 41460417 | A>C | 0.0157 | 2.18 (-8.441,12.801) | 0.6855 | 0.9295 |
| rs117730394 | 3 | 41369232 | G>A | 0.0409 | 1.69 (-6.590,9.962) | 0.6877 | 0.9295 |
| rs56132555 | 3 | 41748278 | C>T | 0.0409 | 1.71 (-6.720,10.145) | 0.6886 | 0.9295 |
| rs147664074 | 3 | 41526484 | T>C | 0.022 | -2.23 (-13.218,8.762) | 0.6892 | 0.9295 |
| rs1691982 | 3 | 41580510 | A>G | 0.4581 | 0.66 (-2.622,3.941) | 0.6917 | 0.9295 |
| rs141634376 | 3 | 41585845 | C>A | 0.0126 | 2.89 (-11.565,17.348) | 0.6931 | 0.9295 |
| rs79077772 | 3 | 41473746 | G>A | 0.0283 | -1.95 (-11.785,7.878) | 0.6950 | 0.9295 |
| rs10514704 | 3 | 41435712 | A>G | 0.0566 | -1.40 (-8.577,5.775) | 0.7001 | 0.9300 |
| rs139092672 | 3 | 41521879 | G>A | 0.022 | -2.05 (-13.023,8.913) | 0.7116 | 0.9419 |
| rs146207490 | 3 | 41425389 | C>T | 0.0535 | 1.21 (-5.660,8.078) | 0.7284 | 0.9585 |
| rs200313692 | 3 | 41302686 | T>C | 0.0221 | 2.02 (-9.642,13.681) | 0.7326 | 0.9585 |
| rs572596217 | 3 | 42012954 | C>G | 0.0189 | -1.98 (-13.728,9.762) | 0.7390 | 0.9585 |
| rs189419948 | 3 | 41919030 | G>A | 0.0346 | 1.32 (-6.827,9.473) | 0.7488 | 0.9585 |
| rs77655392 | 3 | 41397687 | T>G | 0.0157 | -1.91 (-14.828,11.013) | 0.7708 | 0.9585 |
| rs148165660 | 3 | 41848418 | T>C | 0.0157 | -2.05 (-16.397,12.302) | 0.7782 | 0.9585 |
| rs60231851 | 3 | 41549881 | G>A | 0.0157 | -1.84 (-14.764,11.079) | 0.7784 | 0.9585 |
| rs1795316 | 3 | 41531910 | T>G | 0.4591 | 0.48 (-2.960,3.911) | 0.7848 | 0.9585 |
| rs141452813 | 3 | 41398999 | G>A | 0.0157 | -1.76 (-14.884,11.371) | 0.7918 | 0.9585 |
| rs1795338 | 3 | 41587634 | T>C | 0.4591 | 0.46 (-2.978,3.896) | 0.7922 | 0.9585 |
| rs11707955 | 3 | 41308348 | T>C | 0.0975 | 0.72 (-4.931,6.376) | 0.8009 | 0.9585 |
| rs9855102 | 3 | 41775522 | G>A | 0.0346 | 1.14 (-7.932,10.219) | 0.8037 | 0.9585 |
| rs13318330 | 3 | 41812404 | T>C | 0.0346 | 1.14 (-7.932,10.219) | 0.8037 | 0.9585 |
| rs9820447 | 3 | 41848569 | G>A | 0.0346 | 1.14 (-7.932,10.219) | 0.8037 | 0.9585 |
| rs9870782 | 3 | 41903583 | G>A | 0.0346 | 1.14 (-7.932,10.219) | 0.8037 | 0.9585 |
| rs9829586 | 3 | 41920515 | G>C | 0.0346 | 1.14 (-7.932,10.219) | 0.8037 | 0.9585 |
| rs9819366 | 3 | 41948932 | G>A | 0.0346 | 1.14 (-7.932,10.219) | 0.8037 | 0.9585 |
| rs116260485 | 3 | 42029853 | C>T | 0.0346 | 1.14 (-7.932,10.219) | 0.8037 | 0.9585 |
| rs10510718 | 3 | 41369067 | C>T | 0.0849 | 0.74 (-5.120,6.595) | 0.8038 | 0.9585 |
| rs115190709 | 3 | 41386622 | C>T | 0.0849 | 0.75 (-5.259,6.767) | 0.8046 | 0.9585 |
| rs75207131 | 3 | 41539283 | G>A | 0.0786 | -0.82 (-7.406,5.764) | 0.8056 | 0.9585 |
| rs190456313 | 3 | 41373126 | T>A | 0.0157 | -2.02 (-18.278,14.240) | 0.8064 | 0.9585 |
| rs139478908 | 3 | 41547281 | T>C | 0.0314 | 1.12 (-8.083,10.319) | 0.8105 | 0.9585 |
| rs182172903 | 3 | 41800421 | C>A | 0.0157 | -1.63 (-15.018,11.764) | 0.8106 | 0.9585 |
| rs117739398 | 3 | 41864914 | G>A | 0.0157 | 1.52 (-11.227,14.276) | 0.8135 | 0.9585 |
| rs76582599 | 3 | 41761322 | T>C | 0.0157 | 1.53 (-11.314,14.383) | 0.8137 | 0.9585 |
| rs117486137 | 3 | 41904009 | T>C | 0.0157 | 1.53 (-11.314,14.383) | 0.8137 | 0.9585 |
| rs34982278 | 3 | 41955464 | G>A | 0.217 | 0.47 (-3.483,4.417) | 0.8156 | 0.9585 |
| rs78881482 | 3 | 41598227 | C>A | 0.1038 | -0.62 (-5.969,4.730) | 0.8192 | 0.9585 |
| rs117094203 | 3 | 41755113 | C>A | 0.0189 | 1.41 (-11.042,13.853) | 0.8237 | 0.9585 |
| rs142809110 | 3 | 41353128 | C>T | 0.1667 | -0.53 (-5.309,4.251) | 0.8270 | 0.9585 |
| rs9311289 | 3 | 41827063 | G>A | 0.4969 | 0.34 (-2.727,3.406) | 0.8271 | 0.9585 |
| rs372716654 | 3 | 41677354 | T>C | 0.0126 | 1.56 (-12.797,15.919) | 0.8301 | 0.9585 |
| rs147107538 | 3 | 41817633 | C>A | 0.0189 | 1.25 (-10.515,13.019) | 0.8337 | 0.9585 |
| rs76381700 | 3 | 42021495 | C>A | 0.0126 | -1.51 (-15.706,12.695) | 0.8343 | 0.9585 |
| rs12497302 | 3 | 41565428 | T>A | 0.1006 | 0.56 (-4.840,5.965) | 0.8371 | 0.9585 |
| rs35683412 | 3 | 41531759 | C>G | 0.0755 | 0.62 (-5.392,6.632) | 0.8387 | 0.9585 |
| rs146560577 | 3 | 41788304 | T>C | 0.0157 | -1.47 (-15.980,13.036) | 0.8413 | 0.9585 |
| rs78737208 | 3 | 41842540 | G>A | 0.0157 | -1.47 (-15.980,13.036) | 0.8413 | 0.9585 |
| rs145553802 | 3 | 41508816 | G>A | 0.0252 | -1.05 (-12.092,10.000) | 0.8517 | 0.9642 |
| rs12637634 | 3 | 41908893 | G>A | 0.0126 | -1.51 (-17.87,14.848) | 0.8553 | 0.9642 |
| rs191596099 | 3 | 41631806 | T>C | 0.0126 | -1.29 (-15.937,13.351) | 0.8617 | 0.9642 |
| rs182414843 | 3 | 41661389 | C>T | 0.0126 | -1.29 (-15.937,13.351) | 0.8617 | 0.9642 |
| rs187784922 | 3 | 41743793 | C>T | 0.0126 | -1.29 (-15.937,13.351) | 0.8617 | 0.9642 |
| rs142988060 | 3 | 41963053 | G>A | 0.0377 | 0.71 (-7.717,9.146) | 0.8672 | 0.9642 |
| rs143210593 | 3 | 41882862 | G>A | 0.0364 | 0.74 (-8.445,9.922) | 0.8738 | 0.9642 |
| rs75965826 | 3 | 41474867 | G>A | 0.0409 | 0.61 (-7.579,8.803) | 0.8828 | 0.9642 |
| rs73069293 | 3 | 41974223 | G>C | 0.0629 | -0.51 (-7.372,6.352) | 0.8834 | 0.9642 |
| rs190755910 | 3 | 41850431 | C>T | 0.0157 | -0.96 (-13.827,11.914) | 0.8834 | 0.9642 |
| rs188048257 | 3 | 41292968 | T>C | 0.0126 | 1.09 (-13.838,16.020) | 0.8853 | 0.9642 |
| rs116952745 | 3 | 41581943 | G>T | 0.0472 | -0.58 (-8.582,7.418) | 0.8858 | 0.9642 |
| rs1795311 | 3 | 41560191 | T>C | 0.1069 | 0.39 (-4.963,5.742) | 0.8858 | 0.9642 |
| rs117108138 | 3 | 41807390 | G>A | 0.0126 | -0.98 (-15.149,13.197) | 0.8919 | 0.9668 |
| rs542843671 | 3 | 41947999 | T>A | 0.0126 | -0.96 (-15.412,13.486) | 0.8953 | 0.9668 |
| rs1386601 | 3 | 41420792 | A>C | 0.456 | 0.19 (-2.973,3.347) | 0.9069 | 0.9668 |
| rs142092945 | 3 | 41424439 | T>C | 0.0223 | 0.52 (-9.062,10.110) | 0.9141 | 0.9668 |
| rs542949262 | 3 | 41702586 | C>T | 0.0126 | -0.80 (-15.585,13.980) | 0.9147 | 0.9668 |
| rs79200603 | 3 | 41613901 | C>T | 0.0881 | 0.32 (-5.551,6.188) | 0.9147 | 0.9668 |
| rs33916626 | 3 | 41494618 | C>T | 0.0126 | -0.80 (-15.693,14.099) | 0.9159 | 0.9668 |
| rs4246670 | 3 | 42040966 | C>T | 0.2013 | 0.21 (-3.990,4.418) | 0.9199 | 0.9668 |
| rs17210774 | 3 | 41527534 | C>T | 0.0157 | 0.67 (-12.686,14.022) | 0.9214 | 0.9668 |
| rs73075193 | 3 | 41540555 | C>G | 0.0157 | 0.67 (-12.686,14.022) | 0.9214 | 0.9668 |
| rs13089080 | 3 | 41550456 | A>G | 0.0157 | 0.67 (-12.686,14.022) | 0.9214 | 0.9668 |
| rs10510720 | 3 | 41563441 | A>G | 0.0157 | 0.67 (-12.686,14.022) | 0.9214 | 0.9668 |
| rs140994888 | 3 | 41537834 | T>G | 0.0283 | 0.47 (-9.280,10.225) | 0.9239 | 0.9668 |
| rs117185643 | 3 | 41328874 | T>C | 0.0126 | 0.63 (-13.547,14.799) | 0.9305 | 0.9710 |
| rs80131009 | 3 | 41910817 | G>A | 0.0252 | 0.36 (-10.015,10.735) | 0.9454 | 0.9807 |
| rs9883023 | 3 | 41716363 | G>C | 0.0409 | -0.28 (-8.773,8.212) | 0.9480 | 0.9807 |
| rs149557857 | 3 | 41416864 | C>T | 0.038 | 0.21 (-8.162,8.589) | 0.9599 | 0.9892 |
| rs149274676 | 3 | 41470981 | G>C | 0.0157 | 0.33 (-13.230,13.882) | 0.9621 | 0.9892 |
| rs150504687 | 3 | 42034793 | G>C | 0.0157 | -0.29 (-13.182,12.600) | 0.9644 | 0.9892 |
| rs13074845 | 3 | 41472561 | T>C | 0.4811 | -0.06 (-3.316,3.194) | 0.9706 | 0.9898 |
| rs2029254 | 3 | 41456748 | A>G | 0.4874 | 0.05 (-2.931,3.025) | 0.9751 | 0.9917 |
| rs180891216 | 3 | 41866160 | A>C | 0.0252 | 0.11 (-10.113,10.327) | 0.9835 | 0.9946 |
| rs78328636 | 3 | 41587818 | C>T | 0.0755 | -0.06 (-6.920,6.806) | 0.9870 | 0.9946 |
| rs11922599 | 3 | 41598719 | A>C | 0.4717 | 0.02 (-3.418,3.465) | 0.9891 | 0.9946 |
| rs74838353 | 3 | 41461819 | C>T | 0.0503 | 0.00 (-7.010,7.010) | 0.9999 | 0.9999 |

SNP indicates single nucleotide polymorphism; MAF: Minor Allele Frequency; q-FDR: False Discovery Rate q-values.

Models adjusted for sex, smoking status, hypertension controlling and renal function.

Data source: TBAD cohort.

**Table S3: Single nucleotide polymorphisms (SNPs) associated with ULK4 gene expression in the European population.**

| **SNP** | **Chr** | **Position** | **A1/A2** | **Probe** | **Gene** | **SNP with Expression** | | | **SNP with AD** | | |
| --- | --- | --- | --- | --- | --- | --- | --- | --- | --- | --- | --- |
|  |  |  |  |  |  | **Beta** | **SE** | ***P*** | **Beta** | **SE** | ***P*** |
| rs1717022 | 3 | 41984041 | A/G | ENSG00000168038 | ULK4 | 0.42 | 0.042 | <0.0001 | -3.42 | 1.686 | 0.0446 |
| rs11720271 | 3 | 41852434 | G/A | ENSG00000168038 | ULK4 | -0.33 | 0.083 | <0.0001 | -8.39 | 4.251 | 0.0504 |
| rs62258621 | 3 | 41867474 | G/A | ENSG00000168038 | ULK4 | -0.33 | 0.053 | <0.0001 | -8.39 | 4.251 | 0.0504 |
| rs13084436 | 3 | 42017537 | A/T | ENSG00000182606 | ULK4 | 0.92 | 0.04 | <0.0001 | -2.71 | 1.953 | 0.1678 |
| rs7616123 | 3 | 41798941 | G/A | ENSG00000157093 | ULK4 | 0.83 | 0.037 | <0.0001 | -2.65 | 2.043 | 0.1965 |
| rs9810731 | 3 | 41882101 | T/C | ENSG00000168038 | ULK4 | 1.11 | 0.046 | <0.0001 | -2.62 | 2.042 | 0.2023 |
| rs6763508 | 3 | 41750989 | C/T | ENSG00000168038 | ULK4 | 0.83 | 0.038 | <0.0001 | -1.98 | 2.055 | 0.3363 |
| rs60659132 | 3 | 41678906 | T/C | ENSG00000168038 | ULK4 | -0.28 | 0.06 | <0.0001 | 1.39 | 1.758 | 0.4302 |
| rs77212658 | 3 | 41601916 | C/T | ENSG00000168038 | ULK4 | -0.25 | 0.048 | <0.0001 | -2.30 | 3.018 | 0.4478 |
| rs1716699 | 3 | 41898272 | C/T | ENSG00000168038 | ULK4 | -0.37 | 0.061 | <0.0001 | 1.06 | 2.016 | 0.5999 |
| rs9869446 | 3 | 41851210 | G/A | ENSG00000168038 | ULK4 | -0.35 | 0.06 | <0.0001 | 1.07 | 2.032 | 0.6008 |
| rs9855102 | 3 | 41775522 | A/G | ENSG00000182606 | ULK4 | 0.40 | 0.095 | <0.0001 | 1.14 | 4.590 | 0.8037 |
| rs13318330 | 3 | 41812404 | C/T | ENSG00000157093 | ULK4 | 0.38 | 0.095 | <0.0001 | 1.14 | 4.590 | 0.8037 |
| rs9820447 | 3 | 41848569 | A/G | ENSG00000168038 | ULK4 | 0.39 | 0.097 | <0.0001 | 1.14 | 4.590 | 0.8037 |
| rs9870782 | 3 | 41903583 | A/G | ENSG00000168038 | ULK4 | 0.39 | 0.097 | <0.0001 | 1.14 | 4.590 | 0.8037 |
| rs9829586 | 3 | 41920515 | C/G | ENSG00000168038 | ULK4 | 0.39 | 0.097 | <0.0001 | 1.14 | 4.590 | 0.8037 |
| rs9819366 | 3 | 41948932 | A/G | ENSG00000168038 | ULK4 | 0.39 | 0.097 | <0.0001 | 1.14 | 4.590 | 0.8037 |
| rs143210593 | 3 | 41882862 | A/G | ENSG00000168038 | ULK4 | 0.39 | 0.097 | <0.0001 | 0.74 | 4.643 | 0.8738 |
| rs9883023 | 3 | 41716363 | C/G | ENSG00000182606 | ULK4 | 0.33 | 0.083 | <0.0001 | -0.28 | 4.296 | 0.9480 |

Chr, chromosome; A1, effect allele; A2, alternative allele; EAF, frequency of the effect allele;

Data source: PsychENCODE; TBAD cohort.

SNP with Expression means summary data of significant SNPs which screened by linear regression from PsychENCODE.

SNP with AD means linear regression model outcome of significant SNPs from TBAD cohort.

**Table S4: Single nucleotide polymorphisms (SNPs) associated with all CpG sites of ULK4 gene in the European population.**

| **SNP** | **Chr** | **Position** | **A1/A2** | **CpG** | **CpG_Chr** | **CpG_ Position** | **Gene** | **SNP with Methylation** | | | **SNP with AD** | | |
| --- | --- | --- | --- | --- | --- | --- | --- | --- | --- | --- | --- | --- | --- |
|  |  |  |  |  |  |  |  | **Beta** | **SE** | ***P*** | **Beta** | **SE** | ***P*** |
| rs9810731 | 3 | 41882101 | T>C | cg02108620 | 3 | 42002230 | ULK4 | -0.425 | 0.045 | <0.0001 | -2.616 | 2.042 | 0.2023 |
| rs1716979 | 3 | 41961168 | C>T | cg02648746 | 3 | 42004678 | ULK4 | 0.181 | 0.034 | <0.0001 | -3.232 | 1.676 | 0.0557 |
| rs9810731 | 3 | 41882101 | T>C | cg02648746 | 3 | 42004678 | ULK4 | -0.458 | 0.045 | <0.0001 | -2.616 | 2.042 | 0.2023 |
| rs116260485 | 3 | 42029853 | T>C | cg02648746 | 3 | 42004678 | ULK4 | -0.715 | 0.15 | <0.0001 | 1.143 | 4.59 | 0.8037 |
| rs9810731 | 3 | 41882101 | T>C | cg03022575 | 3 | 42003672 | ULK4 | 0.435 | 0.043 | <0.0001 | -2.616 | 2.042 | 0.2023 |
| rs9810731 | 3 | 41882101 | T>C | cg04010712 | 3 | 42003234 | ULK4 | -0.248 | 0.045 | <0.0001 | -2.616 | 2.042 | 0.2023 |
| rs6763508 | 3 | 41750989 | C>T | cg05589743 | 3 | 41997020 | ULK4 | -0.237 | 0.045 | <0.0001 | -1.982 | 2.055 | 0.3363 |
| rs13084436 | 3 | 42017537 | T>A | cg05590619 | 3 | 42056814 | ULK4 | 0.306 | 0.05 | <0.0001 | -2.708 | 1.953 | 0.1678 |
| rs9810731 | 3 | 41882101 | T>C | cg17473727 | 3 | 41372030 | ULK4 | 0.184 | 0.045 | <0.0001 | -2.616 | 2.042 | 0.2023 |
| rs6763756 | 3 | 41369049 | T>C | cg17473727 | 3 | 41372030 | ULK4 | 0.178 | 0.033 | <0.0001 | -1.870 | 1.561 | 0.233 |
| rs9810731 | 3 | 41882101 | T>C | cg19010139 | 3 | 41695019 | ULK4 | 0.378 | 0.045 | <0.0001 | -2.616 | 2.042 | 0.2023 |
| rs2888057 | 3 | 41441627 | A>G | cg20724032 | 3 | 41460736 | ULK4 | -0.346 | 0.05 | <0.0001 | -8.28 | 3.254 | 0.012 |
| rs2029254 | 3 | 41456748 | A>G | cg20724032 | 3 | 41460736 | ULK4 | 0.127 | 0.032 | <0.0001 | 0.047 | 1.506 | 0.9751 |
| rs1716979 | 3 | 41961168 | C>T | cg22222413 | 3 | 42066796 | ULK4 | -0.139 | 0.034 | <0.0001 | -3.232 | 1.676 | 0.0557 |
| rs13084436 | 3 | 42017537 | T>A | cg22222413 | 3 | 42066796 | ULK4 | 0.691 | 0.05 | <0.0001 | -2.708 | 1.953 | 0.1678 |
| rs62257301 | 3 | 42048851 | A>G | cg25209153 | 3 | 42093455 | ULK4 | 0.208 | 0.041 | <0.0001 | -7.886 | 3.448 | 0.0237 |
| rs1716979 | 3 | 41961168 | C>T | cg25209153 | 3 | 42093455 | ULK4 | 0.254 | 0.034 | <0.0001 | -3.232 | 1.676 | 0.0557 |
| rs10510726 | 3 | 41724117 | G>A | cg25209153 | 3 | 42093455 | ULK4 | -0.605 | 0.104 | <0.0001 | -3.534 | 2.161 | 0.1043 |
| rs72864992 | 3 | 41396066 | A>T | cg25209153 | 3 | 42093455 | ULK4 | 0.185 | 0.047 | <0.0001 | 4.942 | 3.463 | 0.1558 |
| rs13084436 | 3 | 42017537 | T>A | cg25209153 | 3 | 42093455 | ULK4 | -1.275 | 0.045 | <0.0001 | -2.708 | 1.953 | 0.1678 |
| rs1495699 | 3 | 41532752 | G>A | cg25209153 | 3 | 42093455 | ULK4 | 0.189 | 0.033 | <0.0001 | -2.359 | 2.348 | 0.3168 |
| rs60659132 | 3 | 41678906 | T>C | cg25209153 | 3 | 42093455 | ULK4 | 0.183 | 0.044 | <0.0001 | 1.391 | 1.758 | 0.4302 |
| rs1256353 | 3 | 41628218 | C>T | cg25209153 | 3 | 42093455 | ULK4 | 0.201 | 0.047 | <0.0001 | 2.111 | 2.689 | 0.4338 |
| rs994439 | 3 | 41716317 | G>A | cg25209153 | 3 | 42093455 | ULK4 | -0.133 | 0.033 | <0.0001 | -1.31 | 1.745 | 0.4539 |
| rs7629070 | 3 | 41440406 | T>C | cg25209153 | 3 | 42093455 | ULK4 | -0.209 | 0.037 | <0.0001 | -1.312 | 3.132 | 0.676 |

Data source: risbane Systems Genetics Study and Lothian Birth Cohorts of 1921 and 1936; TBAD cohort.

SNP with Methylation means summary data of significant SNPs which screened by linear regression from Brisbane Systems Genetics Study and Lothian Birth Cohorts of 1921 and 1936.

SNP with AD means linear regression model outcome of significant SNPs from TBAD cohort.

**Table S5: Results of Mendelian Randomisation (MR) between gene expression, DNA methylation and onset time of aortic dissection using multiple IV analysis.**

| **Exposure** | **Probe** | **Gene** | **nIV** | **IVs** | **Method** | **Beta** | **SE** | ***P*** |
| --- | --- | --- | --- | --- | --- | --- | --- | --- |
| Expression | ENSG00000168038 | ULK4 | 3 | rs1717022; rs7616123;rs60659132 | Inverse variance weighted (fixed effects) | 4.58 | 1.992 | 0.0214 |
| Expression | ENSG00000168038 | ULK4 | 3 | rs1717022; rs7616123;rs60659132 | Weighted median | 3.96 | 2.121 | 0.0622 |
| Expression | ENSG00000168038 | ULK4 | 3 | rs1717022; rs7616123;rs60659132 | Weighted mode | 3.42 | 2.386 | 0.2880 |
| Expression | ENSG00000168038 | ULK4 | 3 | rs1717022; rs7616123;rs60659132 | Simple mode | 4.40 | 3.454 | 0.3312 |
| Expression | ENSG00000168038 | ULK4 | 3 | rs1717022; rs7616123;rs60659132 | MR Egger | 1.45 | 4.798 | 0.8130 |
| DNA methylation | cg25209153 | ULK4 | 8 | rs9852315; rs72864992; rs1495699; rs1256353; rs60659132; rs10510726; rs9810731; rs1716979 | Inverse variance weighted (fixed effects) | -4.02 | 1.662 | 0.0155 |
| DNA methylation | cg25209153 | ULK4 | 8 | rs9852315; rs72864992; rs1495699; rs1256353; rs60659132; rs10510726; rs9810731; rs1716979 | Weighted median | -3.74 | 1.939 | 0.0538 |
| DNA methylation | cg25209153 | ULK4 | 8 | rs9852315; rs72864992; rs1495699; rs1256353; rs60659132; rs10510726; rs9810731; rs1716979 | Simple mode | -9.50 | 4.133 | 0.0612 |
| DNA methylation | cg25209153 | ULK4 | 8 | rs9852315; rs72864992; rs1495699; rs1256353; rs60659132; rs10510726; rs9810731; rs1716979 | Weighted mode | -3.38 | 1.919 | 0.1284 |
| DNA methylation | cg25209153 | ULK4 | 8 | rs9852315; rs72864992; rs1495699; rs1256353; rs60659132; rs10510726; rs9810731; rs1716979 | MR Egger | -2.23 | 2.737 | 0.4517 |
| DNA methylation | cg22222413 | ULK4 | 1 | rs1716979 | Wald ratio | 23.19 | 12.019 | 0.0537 |
| DNA methylation | cg17473727 | ULK4 | 2 | rs6763756; rs9810731 | Inverse variance weighted | 11.92 | 6.878 | 0.0830 |
| DNA methylation | cg02648746 | ULK4 | 3 | rs116260485; rs1716979; rs9810731 | Inverse variance weighted | -5.30 | 4.175 | 0.2040 |
| DNA methylation | cg02648746 | ULK4 | 3 | rs116260485; rs1716979; rs9810731 | MR Egger | 5.38 | 7.294 | 0.5957 |
| DNA methylation | cg02648746 | ULK4 | 3 | rs116260485; rs1716979; rs9810731 | Weighted median | -4.34 | 3.877 | 0.2627 |
| DNA methylation | cg02648746 | ULK4 | 3 | rs116260485; rs1716979; rs9810731 | Simple mode | -3.57 | 5.667 | 0.5929 |
| DNA methylation | cg02648746 | ULK4 | 3 | rs116260485; rs1716979; rs9810731 | Weighted mode | -4.06 | 4.614 | 0.4720 |
| DNA methylation | cg02108620 | ULK4 | 1 | rs9810731 | Wald ratio | -6.16 | 4.810 | 0.2002 |
| DNA methylation | cg03022575 | ULK4 | 1 | rs9810731 | Wald ratio | 6.01 | 4.695 | 0.2002 |
| DNA methylation | cg04010712 | ULK4 | 1 | rs9810731 | Wald ratio | -10.54 | 8.226 | 0.2002 |
| DNA methylation | cg19010139 | ULK4 | 1 | rs9810731 | Wald ratio | 6.93 | 5.410 | 0.2002 |
| DNA methylation | cg20724032 | ULK4 | 2 | rs2029254; rs2888057 | Inverse variance weighted | -14.60 | 11.825 | 0.2171 |
| DNA methylation | cg05589743 | ULK4 | 1 | rs6763508 | Wald ratio | -8.38 | 8.684 | 0.3346 |

Data source: TBAD cohort; PsychENCODE; Brisbane Systems Genetics Study; Lothian Birth Cohorts.

nSNP means the number of significant SNPs which screened by QTL summary data with Linkage Disequilibrium clumping($r^{2}<0.2$).

**Figure S1: Quality Control Procedure for Sequencing Data**


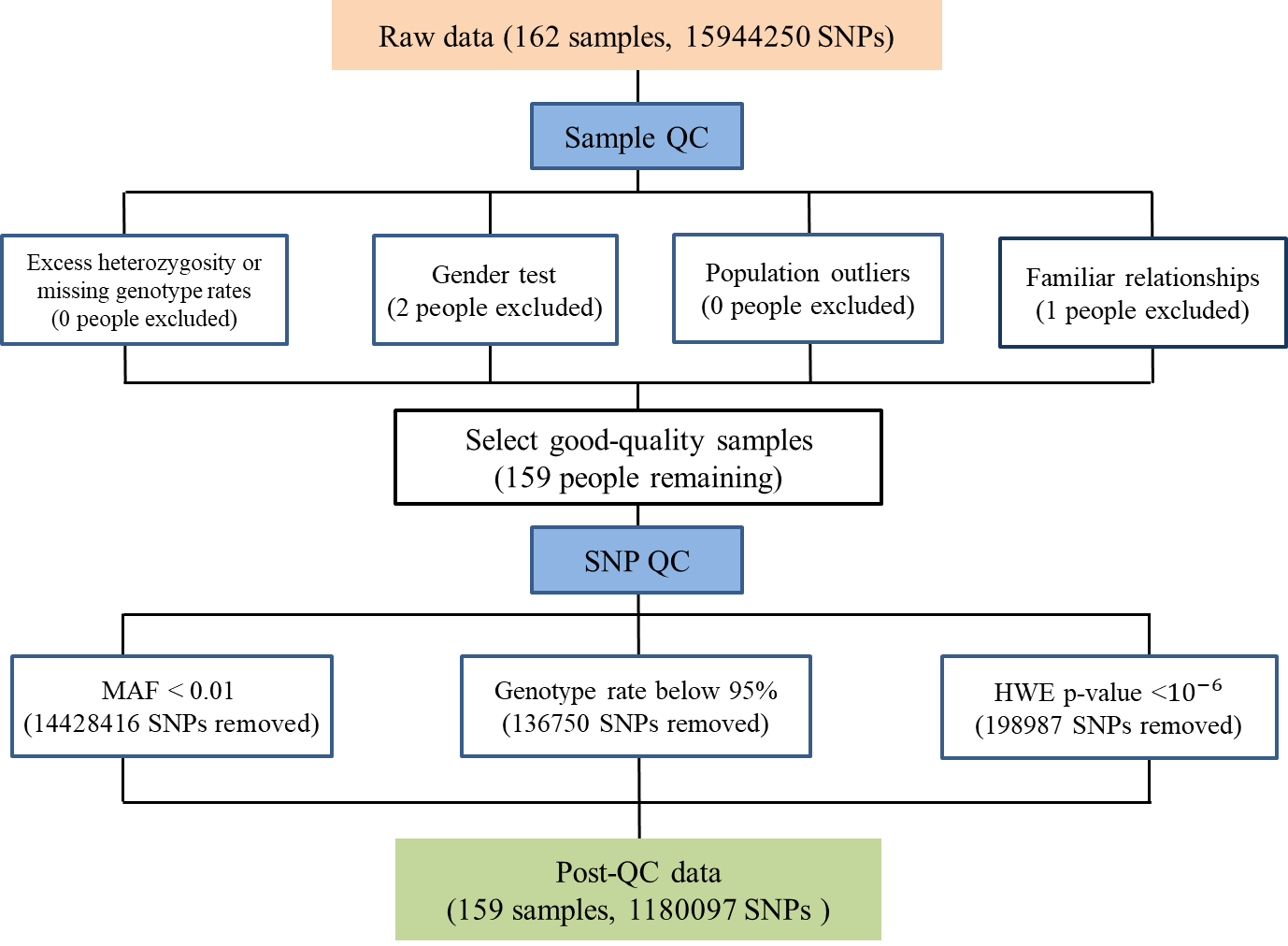


**Figure S2: Regional and LD plots for ULK4**

**(A) Regional plot of candidate region at 40.75 – 42.46 Mb on the position for ULK4.**

**(B) LD block for the region located at 40.75 – 42.46 Mb on ULK4.**


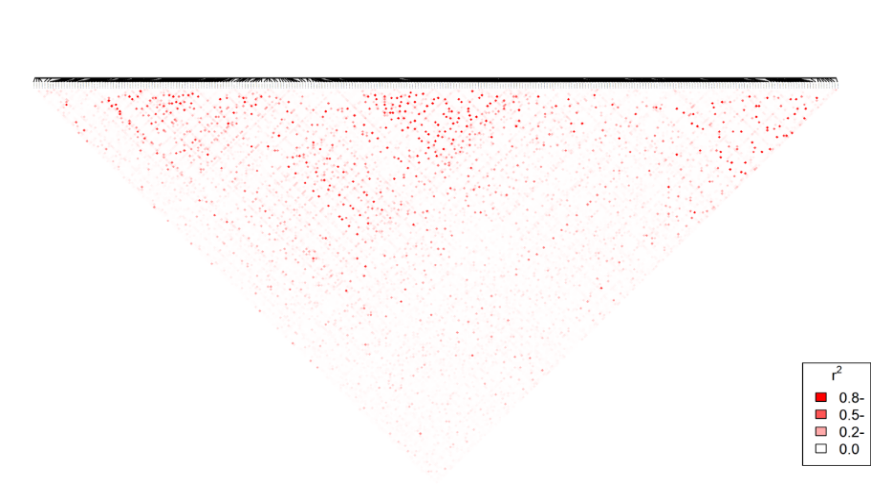

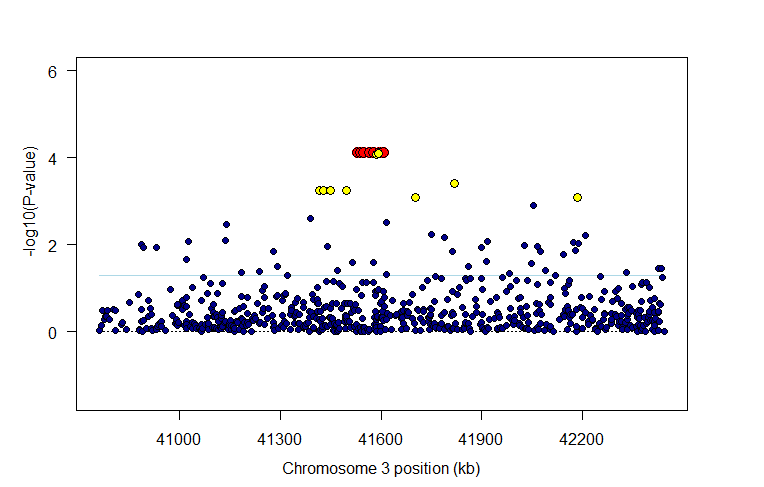


A

B

**Figure S3: Subgroup analyses on genetic score of significant SNPs in ULK4 gene by clinical features**

**
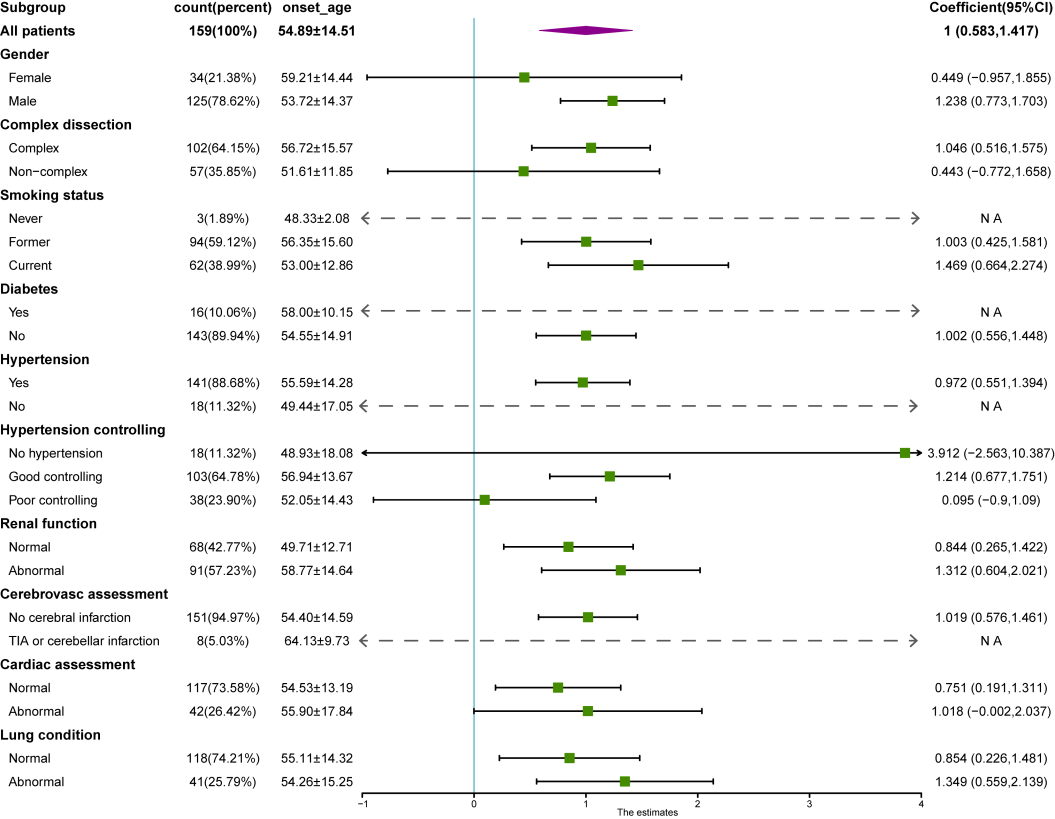
**

**Figure S4: Bioinformatics analyses results**

**(A) Gene Ontology (GO) enrichment analysis of ULK family. (B) KEGG pathway enrichment of ULK family.**

**(C) Gene coexpression network of ULK4. (D) Gene Ontology (GO) enrichment analysis of ULK4 co-expressed genes.**

The significance level of enrichment was set at corrected p-value (q-value) < 0.05.

**
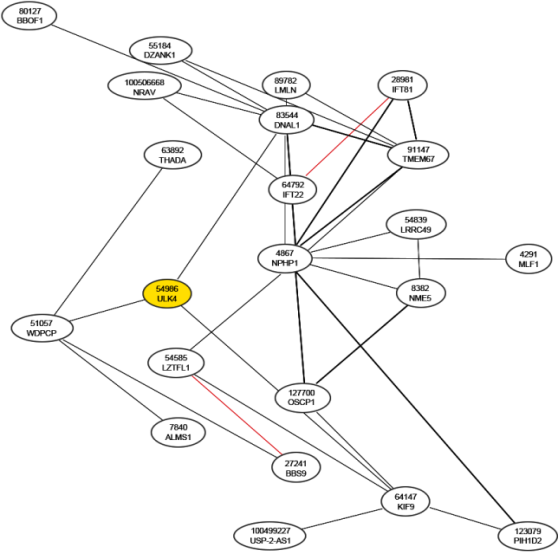

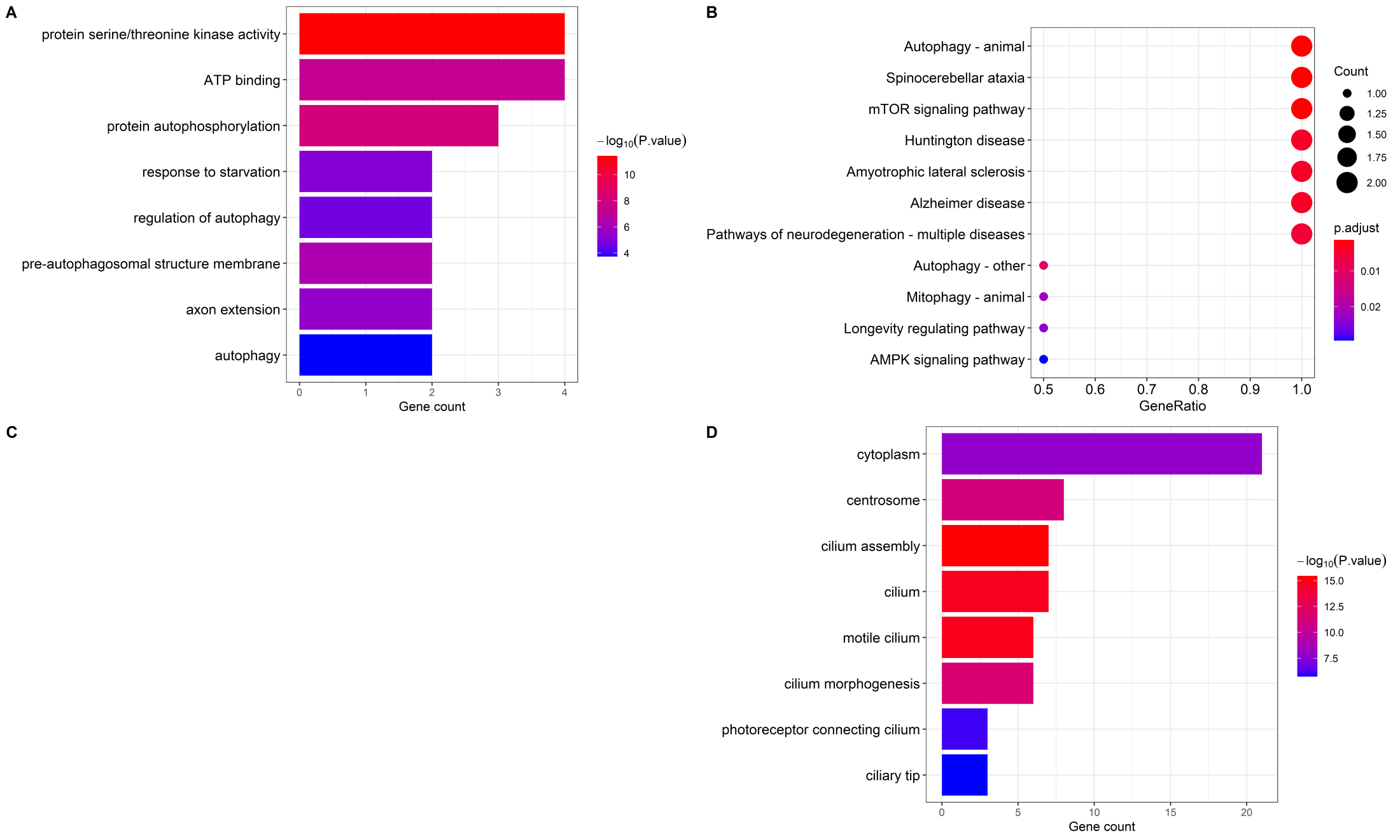
**

The significance level of enrichment was set at corrected p-value (q-value) < 0.05.
